# A target product profile and roadmap for Japanese encephalitis diagnostics in endemic areas in the context of other flaviviruses

**DOI:** 10.1371/journal.pntd.0014683

**Published:** 2026-09-08

**Authors:** Tehmina Bharucha, Laura Stokes, Dorcas Anane-Amponsah, Asha Mary Abraham, Prasert Auewarakul, Balkrishna Awal, Ashwin Y. Belludi, Vijay Bondre, Tina Damodar, Xavier de Lamballerie, Rahul Dhodapkar, Audrey Dubot-Pérès, Veasna Duong, Ava Easton, Roger Evans, Varja Grabovac, Nivedita Gupta, Nguyen Hoang Thien Huong, Siraj Ahmed Khan, Uddhava V. Kinhal, Kaveri Krishnasamy, Chang-Kweng Lim, Lonika Lodha, Gathsaurie Neelika Malavige, Shoba Mammen, Mayfong Mayxay, Angel Miraclin T., Labanya Mukhopadhyay, Mick Mulders, Ashok Munivenkatappa, Adrian Keith Noronha, Mong How Ooi, Ashley Otter, Paul Pasco, Sakib Akther Pattassery, Shruti Radera, Vykuntaraju K. Gowda, Vijayalakshmi Reddy, Priscilla Rupali, Kavitha Saravu, Lucky Sangal, Sanyalack Saysanasongkham, Sadia Shakoor, Krishna Srinavasan, Sharmin Sultana, Aswini M. A., Bhagteshwar Singh, Tom Solomon, Ava Kristy S. Y., Le Van Tan, Kevin K. A. Tetteh, Trieu Huynh Trung, Lance Turtle, Ravi Vasanthapuram, Manjunatha M. Venkataswamy, Nguyen Thanh Vu, Robert J. Wilkinson, Sangay Zangmo, Reeta Mani, Sabine Dittrich

**Affiliations:** 1 The Francis Crick Institute, London, United Kingdom; 2 Department of Infectious Diseases, Imperial College Healthcare NHS Trust, London, United Kingdom; 3 Department of Infectious Diseases, Imperial College London, London, United Kingdom; 4 London School of Hygiene & Tropical Medicine (LSHTM), London, United Kingdom; 5 Deggendorf Institute of Technology, Deggendorf, Germany; 6 Department of Virology, Christian Medical College, Vellore, India; 7 Emerging Infectious Diseases Research Unit, Research Department, Faculty of Medicine Siriraj Hospital, Mahidol University, Bangkok, Thailand; 8 National Public Health Laboratory, Department of Health Services, Kathmandu, Nepal; 9 Department of Neurovirology, National Institute of Mental Health and Neuroscience (NIMHANS), Bengaluru, India; 10 National Institute of Virology (NIV), Indian Council of Medical Research (ICMR), Pune, India; 11 Unité des Virus Émergents (UVE: Aix-Marseille Univ, Università di Corsica, IRD 190, Inserm, IRBA), Marseille, France; 12 Jawaharlal Institute of Postgraduate Medical Education and Research, Puducherry, India; 13 Lao-Oxford-Mahosot Hospital-Wellcome Trust Research Unit (LOMWRU), Microbiology Laboratory, Mahosot Hospital, Vientiane, Laos; 14 Centre for Tropical Medicine and Global Health, Nuffield Department of Clinical Medicine, University of Oxford, Oxford, United Kingdom; 15 Insitut Pasteur du Cambodge, Phnom Penh, Cambodia; 16 Encephalitis International, Malton, United Kingdom; 17 Department of Clinical Infection, Microbiology & Immunology, Institute of Infection, Veterinary & Ecological Sciences, University of Liverpool, Liverpool, United Kingdom; 18 World Health Organization (WHO), Western Pacific Region, Manila, Philippines; 19 Indian Council of Medical Research (ICMR), New Delhi, India; 20 Pham Ngoc Thach University of Medicine, Ho Chi Minh City, Vietnam; 21 Indian Council of Medical Research (ICMR) - Regional Medical Research Centre, Dibrugarh, India; 22 Department of Paediatric Neurology, Indira Gandhi Institute of Child Health (IGICH), Bengaluru, India; 23 King Institute of Preventive Medicine and Research, Chennai, India; 24 Laboratory of Arboviruses, Department of Virology 1, National Institute of Infectious Diseases, Japan Institute for Health Security (JIHS), Tokyo, Japan; 25 Department of Immunology and Molecular Medicine, Faculty of Medical Sciences, University of Sri Jayewardenepura, Nugegoda, Sri Lanka; 26 Department of Neurosciences, Christian Medical College, Vellore, India; 27 Department of Immunizations, Vaccines and Biologicals, WHO, Geneva, Switzerland; 28 National Institute of Virology (NIV), Indian Council of Medical Research (ICMR), Bengaluru, India; 29 Department of Infectious Diseases, Christian Medical College, Vellore, India; 30 Department of Paediatrics, Sarawak General Hospital, Sarawak, Malaysia; 31 Institute of Health and Community Medicine, Universiti Malaysia Sarawak, Kota Samarahan, Malaysia; 32 UK Health Security Agency (UKHSA), Porton Down, United Kingdom; 33 Division of Adult Neurology, Department of Neurosciences, College of Medicine and Philippine General Hospital, University of the Philippines, Manila, Philippines; 34 Department of Microbiology, King George's Medical University, Lucknow, India; 35 Department of Infectious Diseases, Kasturba Medical College, Manipal Academy of Higher Education, Manipal, India; 36 WHO, South-East Asia Region, New Delhi, India; 37 Department of Emergency Medicine, National Children's Hospital, Faculty of Medicine, University of Health Sciences, Vientiane, Laos; 38 Department of Pathology and Laboratory Medicine, Aga Khan University Hospital, Karachi, Pakistan; 39 Vijayanagar Institute of Medical Sciences, Bellary, Karnataka, India; 40 Department of Virology, Institute of Epidemiology Disease Control & Research (IEDCR), Dhaka, Bangladesh; 41 National Institute for Health and Care Research Health Protection Research Unit in Emerging and Zoonotic Infections, University of Liverpool, Liverpool, United Kingdom; 42 Tropical and Infectious Diseases Unit, Liverpool University Hospitals NHS Foundation Trust, Liverpool, United Kingdom; 43 The Pandemic Institute, Liverpool, United Kingdom; 44 Department of Neurology, Walton Centre NHS Foundation Trust, Liverpool, United Kingdom; 45 Oxford University Clinical Research Unit (OUCRU), Ho Chi Minh City, Vietnam; 46 Foundation for Innovative New Diagnostics (FIND), Geneva, Switzerland; 47 Hospital for Tropical Diseases, Ho Chi Minh City, Vietnam; 48 Arbovirus Laboratory, Department of Microbiology and Immunology, Pasteur Institute in Ho Chi Minh city, Ho Chi Minh city, Vietnam; 49 Royal Centre for Disease Control, Ministry of Health, Thimphu, Bhutan; Colorado State University, UNITED STATES OF AMERICA

## Abstract

Japanese encephalitis (JE) virus (JEV) is a leading cause of neurological infection in the Asia-Pacific region with devastating socioeconomic consequences. We aimed to develop a target product profile (TPP) for JE diagnostics in endemic areas in the context of other flaviviruses, to better define improvements needed for patient management and public health. Thematic experts were identified from a scoping review, World Health Organization and Encephalitis International databases, as well as author networks. Initial interviews were performed with selected experts to inform a draft TPP document and start a Delphi process, aiming for consensus of ≥75% on each characteristic. A one-day meeting facilitated in-depth discussion, with breakout groups and electronic voting to develop the final TPP document. In total, 460 participants were invited. Five interviews enabled drafting a TPP. Two iterative surveys were completed by 44 respondents, including clinicians, scientists, and public health experts from 15 countries. A hybrid meeting was attended by 42 participants. Seven priority characteristics were discussed, and consensus was achieved for six; no clear consensus was reached for the preferred test sensitivity and specificity, with 73% agreement. Discussion points and areas of expert consensus were consolidated into a roadmap for development and implementation to guide future work. A diverse group of health professionals agreed that improved diagnostics for JE are needed, and that a priority is a test for patient management that detects JE alongside other relevant flavivirus infections. There was a mandate for a rapid diagnostic test (RDT), and it was suggested that this could be implemented in a two-tiered diagnostic algorithm. The final TPP is presented in the paper; this aims to help define the updates needed to better support patient management and public health interventions, to provide a strategic reference document to drive innovation and improve clarity for JE diagnostic test developers.

## Author summary

Japanese encephalitis virus (JEV) is a mosquito-transmitted virus that can infect the brain with devastating consequences. JEV occurs in 25 countries in the Asia-Pacific region, and represents a leading cause of neurological infection in these areas. A major limiting step in making progress to control the disease is the poor accuracy of existing diagnostic methods, and the lack of a rapid diagnostic test (pregnancy/ COVID-style rapid dipstick method) that could be performed in rural areas without laboratory capacity where JEV exists. We sought to draw on experts to systematically define the requirements ‘target product profile’ of future JEV diagnostic tests. We invited 460 participants from different disciplines and countries, started with online interviews of five participants, drafted a TPP, refined it with two rounds of online surveys and then a hybrid meeting. The final TPP is presented in the paper; this aims to help define the updates needed to better support patient management and public health interventions, to provide a strategic reference document to drive innovation and improve clarity for JE diagnostic test developers.

## Introduction

Historically, Japanese encephalitis (JE) has been one of the most significant neurological infections in Asia, primarily affecting poor, rural communities [1,2]. Current data suggest JE remains a leading cause of neurological infection in Asia, with an estimated incidence of 50,000–150,000 JE cases per year [2]. JE is caused by a neurotropic flavivirus, Japanese encephalitis virus (JEV), that is spread in enzootic cycles between water birds (reservoir host), *Culex* species mosquitoes (vector) and pigs (amplifying host) [3]. Humans are considered accidental or dead-end hosts. JEV infection is largely asymptomatic, with approximately one in 250 people infected developing clinical illness [3]. However, each case of JE can be associated with devastating consequences; 15–30% die and 30–50% experience long-term disability [4]. Families can experience extreme impact from JE, with 20–30% of households suffering sustained debts for many years after the acute illness [5,6].

There is no specific antiviral or immunomodulatory treatment for JE with proven efficacy, but supportive measures can improve outcome [7]. There are several vaccines that have been available for many years [4]. In the last two decades, advances have been made in JEV vaccine programmes, however with JE being a zoonosis, it cannot be eradicated, and sustained vaccine coverage is essential [8]. JEV is endemic in 25 countries in the Asia-Pacific region, with emerging epidemiology such as peri-urban expansion and introductions into new areas including across four territories in Australia in 2022 [9]. Sixteen of twenty-five countries include the JEV vaccine in immunisation schedules, either regionally or nationwide, however many questions remain about vaccine implementation and effectiveness [10].

JE cannot be confirmed solely on clinical presentation, therefore diagnostics are key for both patient management and public health interventions [8,11]. For individual patients, a laboratory-confirmed JE diagnosis can facilitate referral for intensive care support, cessation of unnecessary antibiotics and prognostication. Equally, a negative JE test may trigger further investigation for alternative aetiologies, such as scrub typhus, an increasingly recognised differential diagnosis that is treatable with antibiotics [12]. For public health management, a laboratory-confirmed JE diagnosis supports the selection of the population at risk to vaccinate and ultimately the evaluation of the success of immunisation programmes. In the context of outbreaks, JE confirmation may also lead to mosquito-control measures, such as intermittent irrigation and space spraying of insecticides [13].

However, there are issues with JE diagnostics, highlighted in recent World Health Organization (WHO) reports [14,15]. The pathogen, referring primarily to JEV nucleic acid, is rarely detected in human body fluids, rendering common testing methods, such as molecular diagnosis of JEV nucleic acid, less useful. The mainstay of diagnosis and the WHO recommended test is detection of anti-JEV immunoglobulin M (IgM) by enzyme-linked immunosorbent assay (JEV IgM ELISA) in cerebrospinal fluid (CSF) [16]. While the test procedure takes four to six hours, samples are frequently batched, leading to turnaround times (from sample arriving in the laboratory to result being reported) of several days to weeks. Most importantly, there are issues of poor specificity of JEV ELISA related to non-specific reactivity, persistence of IgM post-vaccination or following previous infection, and cross-reactivity with other endemic flaviviruses (including dengue virus, Zika virus and West Nile virus) [16–18]. Dengue viruses (serotypes 1–4) are endemic in the same areas as JEV, and the epidemiology of other flaviviruses is dynamic and less clear [19]. Lastly, there appears to be increasing reliance on JEV ELISA testing of serum, for which the specificity is lower when compared to the recommended specimen, CSF [16].

The gold standard diagnostic test for JE consists of virus neutralisation tests (VNT), traditionally plaque reduction neutralisation test (PRNT) [16]. VNT is rarely performed in JEV-endemic areas as it is not practical; these tests require paired serum samples with relatively large volumes, cell culture and biosafety containment laboratory 3 facilities, in addition to technical expertise. VNTs typically have a long turnaround time (1–2 weeks), are low-throughput, and are frequently uninterpretable due to endemic flavivirus cross-reactivity of the samples being tested. IgM-VNT, involving immunoglobulin G (IgG) depletion of sera prior to VNT, has been utilised to improve interpretation and may be performed in a single serum sample [20].

This work aimed to engage an interdisciplinary expert group to direct future research and diagnostic development. We developed a target product profile (TPP) for a JE diagnostic test in the context of other endemic flaviviruses, to help define the updates needed to better support patient management and public health interventions. The feedback and discussion points enabled the identification of critical areas to be considered in a roadmap for the use of such a test. The final TPP aims to provide a strategic reference document to drive innovation and improve clarity for JE diagnostic test developers.

## Methods

### Ethics Statement

The study was approved by the Crick Research Ethics Committee (WLK-CREC-2025–51). Verbal consent was obtained from participants for the online interviews.

### Participants

A scoping review was conducted in the PubMed database for relevant literature published over the last ten years from 21/7/15–20/7/25. The search terms included were ‘(Japanese encephalitis) AND diagnos*,’ and results were filtered to include peer-reviewed publications on JE diagnostics in humans. Corresponding authors of relevant articles were contacted to invite them to participate. Participants were also identified from the Southeast Asia Regional Office of WHO (SEARO), Western Pacific Regional Office of WHO (WPRO), and Encephalitis International databases. Additionally, informal professional networks were utilised to ensure a wide variety of expertise in clinical management, public health, and diagnostic implementation from a range of health system contexts.

### TPP process (Fig 1)

- Scoping interviews: A representative sample of participants from diverse disciplines and countries was invited to participate in initial scoping online interviews (which were conducted via Zoom), with the aim of identifying common themes relating to JE diagnostics. The interviews were performed sequentially until data saturation was achieved. These interviews lasted approximately 30–45 minutes, and were conducted by at least two authors (T. Bharucha, L. Stokes, D. Anane-Amponsah and/or S. Dittrich). An interview guide was used to facilitate a semi-structured approach, including questions on 1) the participant’s background, role, and expertise, 2) their perspectives on the existing JE patient and diagnostic pathway, 3) diagnostic needs and test characteristics, and 4) implementation and stakeholder landscape. The information from the interviews, together with published literature reviews of JE diagnostics [16,21], the WHO TPP development document and other published TPPs, informed the development of a preliminary draft JE TPP document [16,22]. The framework follows the WHO TPP development document categories for a diagnostic test, with each individual characteristic having a minimum (minimally acceptable) and preferred profile.
- Delphi process: An iterative Delphi survey was performed, a well-established methodology used to obtain consensus of opinion of a group of experts [23]. Themes that emerged from the online scoping interview were incorporated into the draft TPP and the Delphi survey to obtain feedback from experts on the information needed to support each characteristic. The questions were designed to include a 5-point Likert scale. This rating scale measures strength of agreement/disagreement, with the following choice of answers: strongly disagree, disagree, neutral, agree and strongly agree. The aim was to develop a consensus of ≥75% on each characteristic, defined as ≥75% of participants selecting strongly agree or agree. For each question, there was an opportunity for respondents to provide free-text additional comments. Microsoft Forms software was used to develop the online survey that was sent to all participants identified from the searches. Initial survey feedback after the first round of responses from experts was analysed and used to revise the TPP document incorporating feedback. A follow-up survey was developed with the aim to more closely address user needs and disseminated to assess consensus on the revised content.
- Consensus meeting: Following two Delphi survey rounds, a revised draft TPP document was sent to all participants for review prior to a hybrid consensus meeting. During this hybrid one-day meeting, characteristics with the most disagreement were discussed and voted on to finalise the TPP. Participants engaged in structured discussion through open Q&A sessions, submission of online written feedback, and facilitated breakout group discussions. In addition to the prioritised characteristics, there was an opportunity to discuss the full TPP document. Electronic voting via the Zoom software poll function determined the level of agreement on the final wording of each TPP characteristic, again using the five-point Likert scale ranging from strongly disagree to strongly agree. There was also time allotted for participants to comment on the draft TPP. Following the meeting, the TPP document was circulated to all participants for their final comments.

**Fig 1:**
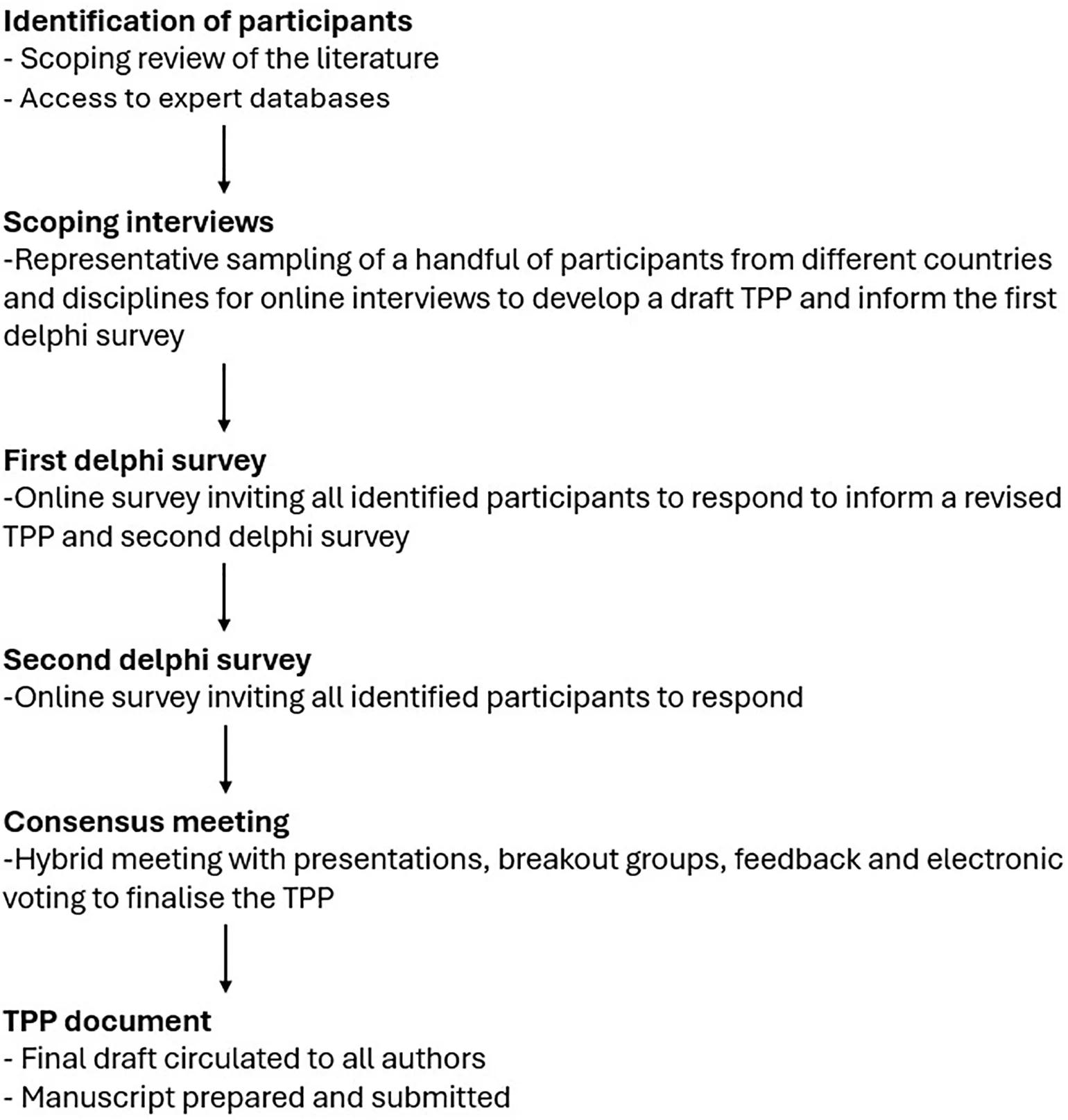
Overview of the TPP process.

### Patient and public involvement

Dr Ava Easton (PhD, Chief Executive of Encephalitis International) acted as a patient advocate for this work.

## Results

### Scoping interviews

Five participants, including clinicians, laboratory scientists and public health experts, each from a different JEV-endemic country were identified and invited for online interviews, all of whom attended. All participants agreed strongly that improved JE diagnostics are needed. Key issues were identified relating to delayed diagnosis, presence of the iceberg phenomenon (where only severe cases are diagnosed), lack of laboratory capacity to perform testing in provincial hospitals, difficulty performing lumbar puncture (LP) and limitations in the sensitivity and specificity of available diagnostics. There was concordance from all participants that novel diagnostics need to be: 1) rapid diagnostic tests (RDTs) to help inform care decisions while at the same time informing public health activities, 2) executed with minimal training, 3) have multiplex testing capabilities for other endemic flaviviruses in order to distinguish cross-reactivity and improve specificity, 4) able to test blood samples in addition to CSF and 5) have higher accuracy. The interviews guided the selection of characteristics included in the Delphi survey, including the purpose of a JE diagnostic test, intended users and setting, clinical performance (sensitivity and specificity), specimen type, and test procedure.

### Consensus building on TPP characteristics

**Fig 2:**
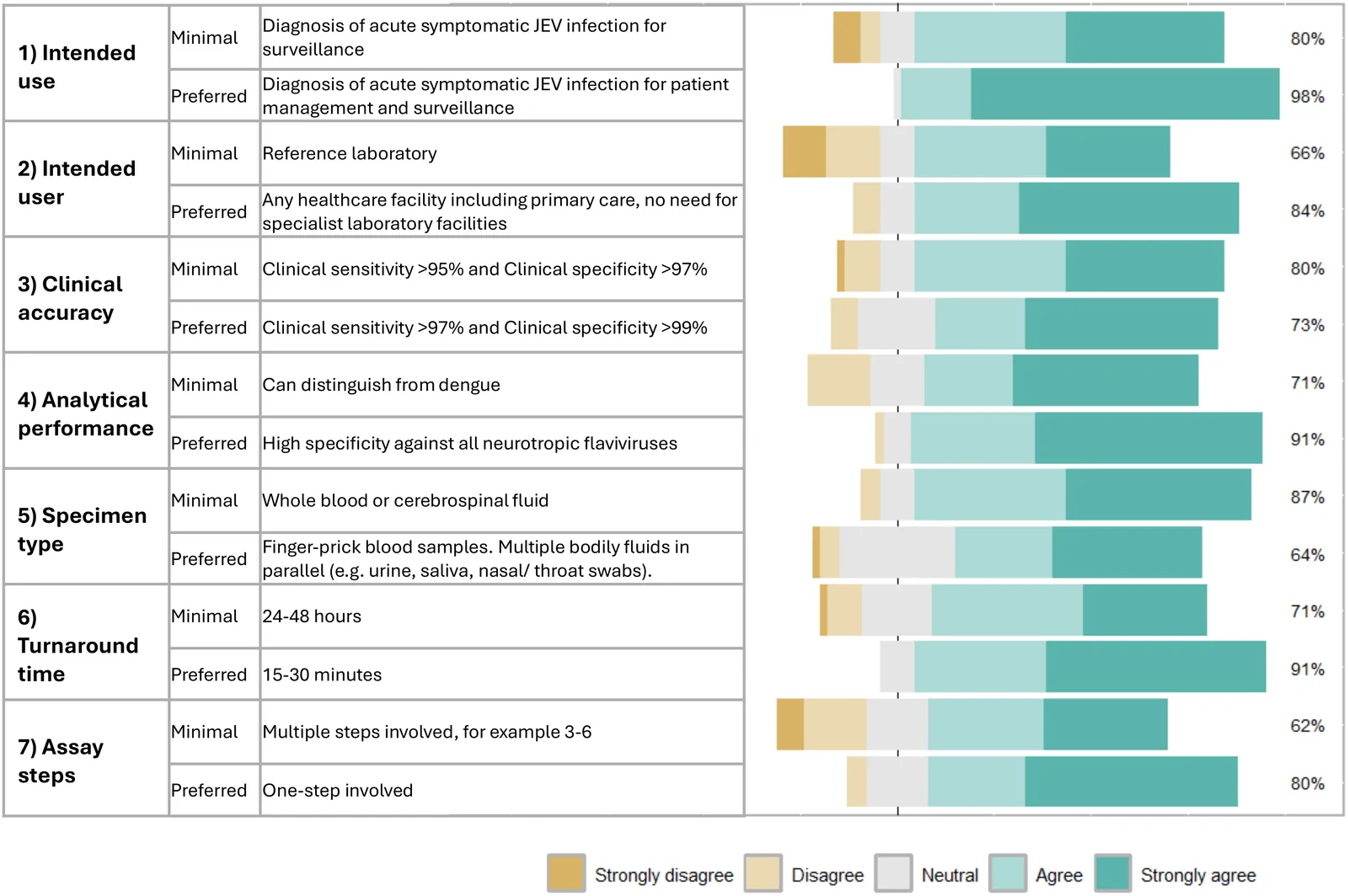
Results of the first Delphi survey.

**Fig 3:**
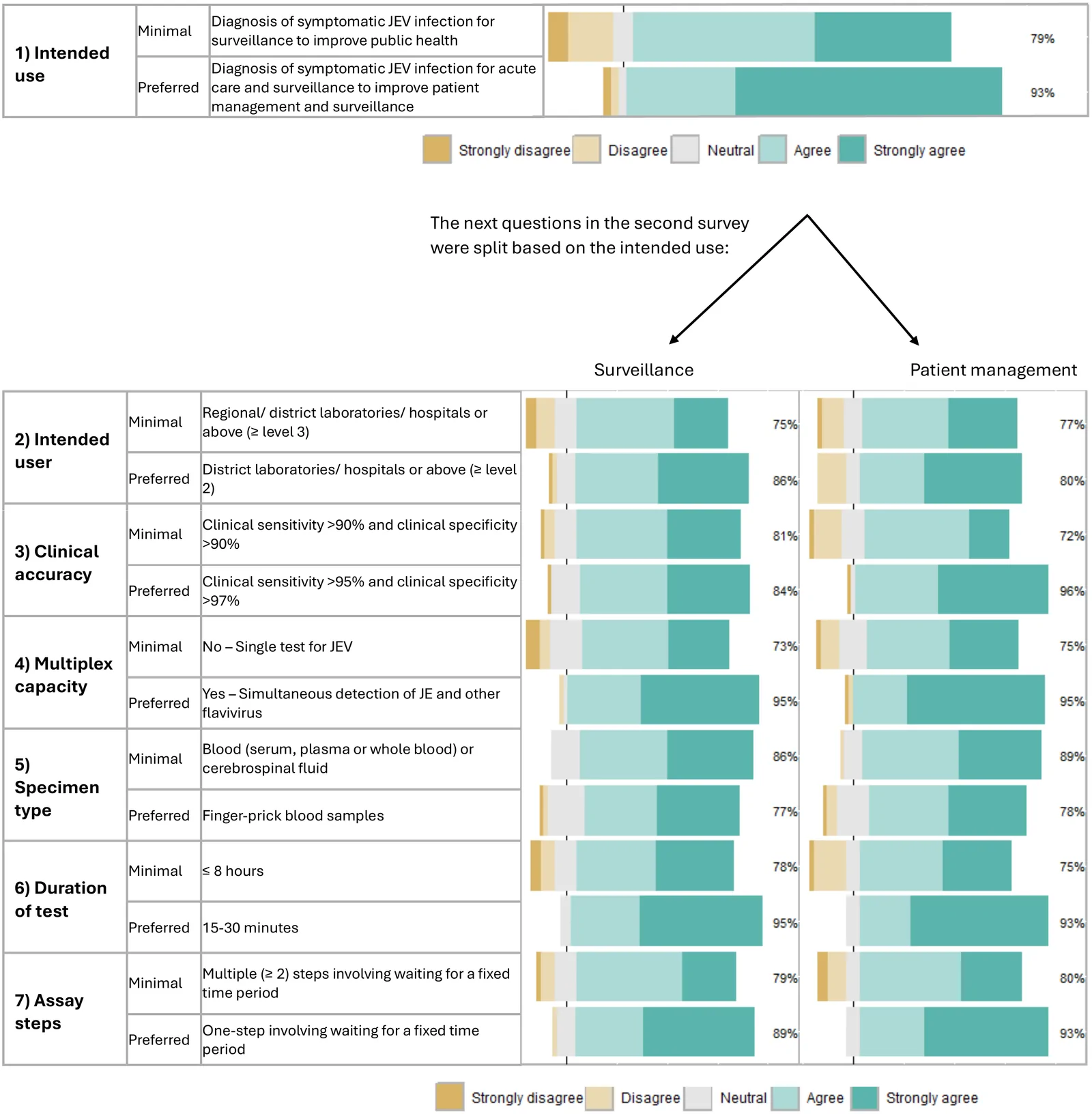
Results of the second Delphi survey.

The TPP was drafted and Microsoft Forms software was used to develop an online survey (‘first survey’, see, S1 Appendix), which was sent to 460 people. The TPP was then edited and an updated online survey (‘second survey’, see, S2 Appendix) was sent to participants. Forty-four (9.6%, 44/460) people responded to the surveys, including clinicians, laboratory scientists, public health experts and policy makers, from sixteen countries (Bangladesh, Bhutan, Cambodia, France, India, Japan, Laos PDR, Malaysia, Nepal, Pakistan, Philippines, Switzerland, Thailand, UK, USA and Vietnam) with a median of 11 years (6–18 interquartile range) of experience working on JE. The aggregated results of the Delphi surveys are displayed in Figs 2 and 3. In the first survey (n = 44), for the minimal profile of a JE diagnostic test, responses demonstrated a lack of agreement on the intended user being in a reference laboratory (66%), turnaround time of 24–48 hours (71%), the test being limited to distinguishing from dengue (71%), and there being multiple [3–6] steps involved (62%). Additional free-text comments emphasised the need to prioritise a test to be used for patient management (72%) and available for point-of-care testing (48%).

In the second survey (n = 44), based on responses in the first survey, the intended use of a JE diagnostic test was separated into patient management and surveillance purposes, with corresponding differences between minimal and preferred requirements. This separation took into account feedback that these purposes needed to be considered as separate entities with unique attributes when addressing the other TPP characteristics. The second survey achieved greater concordance; however, areas of disagreement remained (Fig 3). For example, there were additional free-text comments from 45% of participants that a diagnostic test needed to have multiplexing capacity to detect all endemic flaviviruses as a minimal requirement, and that without this the test would not be sufficiently specific. Also, the minimum sensitivity and specificity values were considered too low for patient management by 27% of participants. Notably, comments from two-thirds of participants in the second survey emphasised the need for novel JE diagnostic tests to prioritise patient management. For this reason, the amended TPP focused on a test for patient management, with the view that any test for patient management will inform surveillance.

### Consensus meeting

A hybrid meeting on 8^th^ October 2025 was attended by 42 participants, and in-person participants met in Bangalore, India.

Prior to the meeting, the revised TPP was shared with all participants. The meeting started with introductions to TPPs and JE diagnostics, hearing from participants in different countries to ensure a common understanding of the challenges and the goal of the meeting.

**Fig 4:**
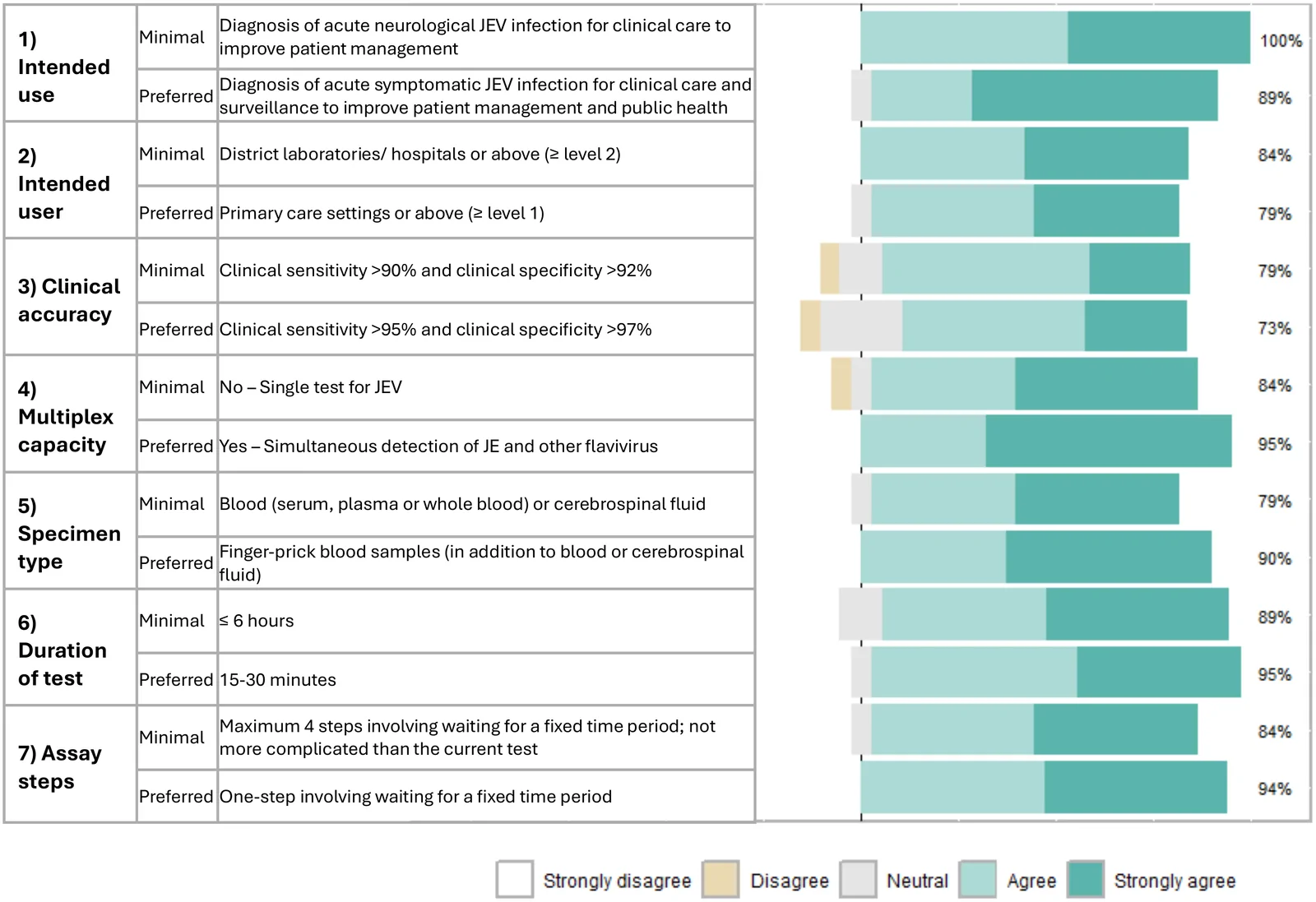
Results of the final Delphi survey performed during the consensus meeting.

Seven priority characteristics were discussed (Fig 4), including an introduction to the TPP characteristic, breakout groups, feedback and finally electronic voting. Consensus was achieved for all except for the preferred test sensitivity and specificity, which achieved 73% agreement.

The final TPP characteristics are presented in Table 1 along with the corresponding discussion points and rationale.

**Table 1:** Final TPP characteristics and rationale.

Category	Characteristic	Minimal	Preferred
**Intended use/** **intended purpose**	**Goal of test/intended use**	**Clinical care to improve patient management** ^**a**^	**Clinical care and surveillance to improve patient management and public health**
	Notes from discussion/ rationale: Previous thinking has been that JE is a vaccine-preventable disease with no specific treatment and that, in view of limited resources, all efforts should be focused on vaccine distribution. There was strong consensus from stakeholders in our TPP process that the priority for the intended use/purpose of a diagnostic test should be for a test that informs patient management. To this end, the *minimal criteria* for the *intended use/intended purpose* is a test for patient management alone, acknowledging that any test for patient management is also likely to inform surveillance. There is no specific treatment for JE that has proven efficacy; however, supportive care such as the management of seizures or raised intracranial pressure does impact on clinical outcome [4,24]. For this reason, a rapid diagnosis of JE and transfer to an intensive care centre (ideally a specialist neuro-intensive care unit) is paramount. For patients, families, and clinicians, a confirmed JE diagnosis is invaluable to manage expectations and prognosticate. Furthermore, if a test is negative, this triggers a search for alternative causes, especially potentially treatable and emerging infections such as scrub typhus. It is also apparent that, in the absence of a real-time rapid diagnostic testing (RDT), it is not possible to even consider treatment trials, a circular dilemma that in the absence of a diagnosis we are not able to identify patient cohorts to evaluate a treatment, and in the absence of a treatment we do not have the impetus to develop a diagnosis for clinical management. JE diagnostics are also crucial for public health, to adequately implement vaccination programmes based on evidence of JE and to evaluate their progress. We know that 16/25 endemic countries have introduced a JEV vaccine into immunisation schedules, however not all are vaccinating nationwide and there are issues with vaccine uptake [16,25]. We need more granular epidemiological data to support introduction of JEV vaccines in other areas, for example in Bangladesh and Pakistan where there is no vaccination programme, and in parts of Malaysia and Indonesia where there is a subnational vaccination programme. There are informal reports of JEV vaccine failure; for example in a recent WHO report, there were issues in Nepal [26]. However, this is difficult to study as patients in these settings frequently have inadequate vaccine records and there are issues with current diagnostics in differentiating between natural infection and previous vaccination. Lastly, there are issues of substandard and falsified vaccines that are poorly recognised, that compound these issues.		
	**Target population**	**Suspected acute symptomatic neurological JE cases (all ages)**	**Suspected acute symptomatic JE cases (all ages)** ^**b**^
	Notes from discussion/ rationale: It is recognised that the spectrum of clinical manifestations of JEV infection is wide, from the large proportion that are asymptomatic, to mild febrile illness to severe neurological disease [27]. The clinical trajectory and outcome of JEV infection presenting as an undifferentiated febrile illness remains poorly understood. It may not be necessary to treat JEV infection without neurological illness. However, for those who do progress to neurological infection, diagnosing JEV infection early in the illness might enable the prompt initiation of supportive neurocritical care or facilitate enrolment in therapeutic clinical trials. Either way, in the absence of these data, considering resource-constrained settings in most JEV-endemic areas, the minimal criteria for defining the target population is the identification of acute symptomatic neurological infection. The diagnostic also needs to cover all ages; following the introduction of JE vaccination (amongst other factors), JE is no longer solely a childhood disease in some areas [9]. In terms of public health surveillance, the focus is also on acute symptomatic neurological JEV infection in all ages in endemic areas. Initially, to implement vaccination programmes in an area, evidence of JEV infection may involve any spectrum of clinical illness, including asymptomatic, acute or previous infection. Existing diagnostics, such as PRNT, are considered and adequately serve the need of demonstrating the presence of sustained JEV transmission to instigate vaccination programmes. However, JEV is a zoonosis, will not be eradicated and requires sustained vaccination programmes. For the evaluation of the success of vaccination programmes, it is important to detect clinically relevant infections that vaccine programmes are trying to prevent, i.e., acute symptomatic neurological infection. Equally, any potential infection needs to be confirmed by reliable methods, differentiating JEV infection from other flavivirus infections and vaccination.		
**Intended user and use setting**	**Target use setting**	**District laboratories/hospitals or above (≥ level 2) [**28,29**]**	**Primary care settings or above (≥ level 1)**
	Notes from discussion/ rationale: There was strong consensus that diagnostic testing needs to be available beyond reference laboratories to improve patient outcomes. Balancing the recognised resource constraints, it was agreed that the minimal requirement was in district laboratories, however being able to diagnose JE in primary care settings was the preferred requirement [22,24]. We used the WHO reference document with a four-tier healthcare system, although we acknowledged that this may not be directly applicable to all healthcare systems, and each individual setting may have its nuances of health service deployment [25].		
	**Target users**	**Trained laboratory technicians [** 30 **]**	**Community health workers without specialised lab training**
	Notes from discussion/ rationale: In view of the paucity of technical expertise, it was agreed that target users should be required to have had only minimal experience in performing laboratory tests, and ideally no training at all. The latter would be achievable with a simple RDT.		
	**User training**	**2-3 days of training required**	**Minimal or no formal training**
	Notes from discussion/ rationale: As above, it was agreed that minimal training (2–3 days) would be feasible, although ideally this would be performed with no formal training at all.		
**Analytical performance**	**Cross-reactivity**	**Minimal cross-reactivity against other endemic flaviviruses**	**Minimal cross-reactivity against other flaviviruses**
	Notes from discussion/ rationale: A priority in the analytical performance was the minimisation of cross-reactivity between different flaviviruses.		
**Clinical Performance**	**Clinical sensitivity**	**≥90%**	**≥95%**
	**Clinical specificity** ^**c**^	**≥92%**	**≥97%**
	Notes from discussion/ rationale: There was no clear consensus reached, with 70% agreement, regarding the sensitivity and specificity of a JE diagnostic test for patient management. There are several factors in choosing these parameters; consideration of what is needed, what might be achievable, and balancing parameters that would make the test clinically useful while not setting the bar too high resulting in failure and discouraging test developers. In terms of what is needed, in view of the fact there is currently no specific treatment, it was suggested that high specificity (ability to rule in a diagnosis of JE) is more useful than high sensitivity (ability to rule out a diagnosis of JE). If JE is falsely diagnosed, clinicians may stop empirical antimicrobials prematurely, or fail to investigate for alternative aetiologies. In terms of achievability, it was accepted that a major constraint in specificity is the cross-reactivity with other flaviviruses, and this may be addressed by stating that any proposed specificity takes into account that contemporaneous testing for other flaviviruses is performed. In terms of not setting ourselves up for failure, we have stated that the metrics are ideal numbers and not an absolute threshold. It is possible that a two-tiered approach, algorithmic testing approach will be needed, in which a highly sensitive RDT is performed. If positive, this could enable further diagnostic testing performed in a local or reference laboratory, referral to a regional hospital, and ultimately treatment trials. It is recognised that there are limitations in our knowledge of the clinical performance of currently used JE tests.		
**Analysis Type**	**Assay targets**	**Pathogen or host biomarkers available to meet the diagnostic needs**	
	Notes from discussion/ rationale: There were no proposed constraints on the assay target, with agreement that this could be a pathogen or host biomarker available to meet the diagnostic needs. It is acknowledged that, although pathogen-based methods such as nucleic acid and protein detection, do not demonstrate high diagnostic yield, there is an important role for them. Aside from improved specificity and potential for tracking mutations and variants, these methods have a role in diagnosing the increasing number of patients with immunosuppression for whom antibody-based methods may not be useful.		
	**Result display and interpretation**	**Interpretation that does not require specialist knowledge [** 31 **]**	**Qualitative (yes/no)**
	Notes from discussion/ rationale: There was agreement that, at a minimum, test interpretation should not require specialist knowledge, and ideally this should be a qualitative (yes/no) display.		
**Specimen type**	**Specimen type**	**Blood (serum, plasma or whole blood) or cerebrospinal fluid (CSF)**	**Finger-prick blood samples (in addition to blood (serum, plasma or whole blood) or CSF)**
	Notes from discussion/ rationale: It has long been recognised that the specificity of the recommended anti-JEV IgM ELISA is poor in serum as opposed to CSF, and that serum should not be relied upon for a diagnosis. Nonetheless, in recent years, there is considerable reliance on serum, and perspectives from the consortia is that CSF is difficult to obtain in all patients, particularly in those with contraindications to LP. For this reason, as a minimum, there was consensus that blood or CSF should be usable. Furthermore, it was preferred for a finger-prick blood sample to be the specimen type used.		
**Test procedure**	**Test format**	**Laboratory-based test**	**Rapid diagnostic test**
	Notes from discussion/ rationale: There was agreement that a laboratory-based test would be a minimal criterion, but that ideally an RDT is needed. The latter will be needed to meet many of the preferred characteristics.		
	**Multiplexing**	**Test for JEV alone**	**Simultaneous detection of JEV and other flaviviruses** ^**d**^
	Notes from discussion/ rationale: There was strong consensus that JEV testing needs to be integrated with testing for other flaviviruses. This arises in particular because of the effect of co-circulation of other flaviviruses on test specificity, as well as overlap in clinical syndromes. It is recognised that there is dynamic epidemiology, and we may well be missing other endemic flaviviruses such as sporadic cases of West Nile virus. While multiplex testing is attractive, it is also associated with increased costs and, if reduced to a single assay there is the potential for reduced sensitivity. Furthermore, a multiplex test useful in one country may not be applicable for use in another. It was agreed that at a minimum, a single JEV test would be useful, but preferably a multiplex test.		
	**Specimen preparation**	**Limited (maximum of 2 steps) specimen preparation**	**No additional steps for sample preparation**
	Notes from discussion/ rationale: It was agreed that the test should require at minimum 2 steps specimen preparation, which might include, for example, centrifugation and addition of a diluent. The preferred criterion was no steps at all, e.g., RDT format using finger-prick blood		
	**Time to result** ^**e**^	**≤6hrs**	**15-30 minuntes**
	Notes from discussion/ rationale: This criterion pertains only to the test procedure, rather than the actual time that the laboratory takes from receiving the sample to providing a result, acknowledging that the two are closely linked. For the existing anti-JEV IgM ELISA, the test procedure is 4 hours, and the agreed minimum criterion was for a similar time, which was 6 hours. However, tests are frequently batched and performed once a week to help maximise use of the existing test kit, leading to increased result turnaround time. In contrast, the preferred criterion is for a RDT performable in 15–30 minutes, and the preferred characteristic for any test is with no laboratory restrictions/ requirements for batching.		
	**Throughput**	**May require testing in batches, for example in a 96-well format**	**Performable as a single test and random-access**
	Notes from discussion/ rationale: Batch testing may be cost-effective, however this may lead to delays in testing and result reporting. Tests that do no require batching are preferred for informing patient management and enrolling into trials.		
	**Ease of use (time steps)**	**Maximum four steps involving waiting for a fixed time period; minimally accepted is not more complicated than current test**^**3**^.	**One step involving waiting for a fixed time period.**
	Notes from discussion/ rationale: The agreed minimal criterion was modelled on the existing test, with a maximum of four steps requiring waiting for a fixed-time period. This is modelled on existing ELISA methods which are the standard diagnostic method in use. The preferred criterion was only one timed step, which would be the number of steps expected in an RDT.		
**Operational Characteristics**	**Test kit**	**Materials to run the test procedure should be included in a kit.**	**One test should be packed individually with all materials required to run a single test procedure. This should include: sample collection devices/equipment (e.g., lancets), reagents, reagent grade water or other consumables. A test kit box should include several individually packaged tests (e.g., 20, 50 or 100 tests) and any other required consumables and controls that are required.**
	Notes from discussion/ rationale: It was agreed that the materials to run the test should be included in the kit. At a minimum, it would be appropriate for standard laboratory consumables to be expected to be available for a laboratory procedure. Preferably, in the case of a RDT, all materials should be included.		
	**Test kit stability/operational conditions**	**Can be stored in refrigerator temperatures, stable at 2–8 °C and 70% humidity. Expiration >18 months; any associated equipment must meet or exceed these requirements [** 32 **]**	**Can be stored at ambient temperatures, stable at 2–40 °C and 90% humidity. Expiration >24 months. Any associated equipment must meet or exceed these requirements.**
	Notes from discussion/ rationale: These criteria are based on previous standards for test kit stability, such as an RDT for SARS-CoV-2 [33]. We acknowledge that desiccants can improve the stability of analytes [34].		
	**Shipping and transport conditions**	**Temperature stress (48h with fluctuations up to 40 °C and down to 2 °C); no cold chain required for transport**	**Temperature stress (72h with fluctuations up to 50 °C and down to 2 °C), no cold chain required for transport, and with indicator of temperature or humidity excursions that would result in invalid or low-performance results.**
	Notes from discussion/ rationale: These criteria are based on previous standards for diagnostic TPPs, with consideration for the environment of regions endemic for JE.		
	**Biosafety**	**Standard safety precautions for specimen collection and processing recommended** [31,35]	
	Notes from discussion/ rationale: These criteria are based on standardised biological safety levels to inform healthcare workers and laboratory personnel about biohazardous risk and appropriate safety precautions [36].		
	**Waste disposal**	**Standard biohazard waste disposal**	**The kit's components are designed to reduce environmental effect during biohazard waste disposal.**
	Notes from discussion/ rationale: These criteria are based on a need for diagnostic tests suitable for use in district/primary healthcare settings, which may not have access to enhanced waste disposal facilities. Standards for safe and sustainable healthcare waste management can prevent adverse health and environmental consequences [37,38].		
	**Kit controls**	**Technical and kit stability controls should be included**^**3**^.	**Same as minimal, plus indicator of inadequate sample and incorrect procedure and/or use.**
	Notes from discussion/ rationale: Inclusion of technical and kit stability controls within a test kit is standard practice, and an essential part of quality control in diagnostic testing, in order to ensure adequate functioning, accuracy and reliability of a test [39]. We would expect that industry standards relevant for specific tests will be fulfilled, for example a control line in any RDT.		
	**Reagent controls**	**Positive and negative controls provided with kit** [31,35]	
	Notes from discussion/ rationale: Notes from discussion/ rationale: inclusion of positive and negative controls in the test kit is standard practice, and an essential part of quality control in diagnostic testing [40].		
	**Sustainability**	**Considerations made to reduce waste and carbon footprint**	
	Notes from discussion/ rationale: The environmental impact of medical diagnostics is becoming increasingly recognised within the sphere of healthcare sustainability, and there is an accepted need for sustainable novel diagnostic technologies [37].		
Pricing and Market Access	**Target list price per test**	**<USD 10** ^**f**^	**<USD 5** ^**f**^
	Notes from discussion/ rationale: It is acknowledged that these tests are intended for use in resource-constrained settings. Nonetheless, this needs to be balanced by the achievability and quality of the test.		

a Acknowledging that any diagnostic test for patient management will inform surveillance

b Including the full spectrum of clinical disease.

c Tested against other endemic neurotropic flaviviruses, e.g., Dengue virus, Zika virus ± West Nile virus.

d JEV and Dengue multiplex test as a priority, or multiplex test for neurological infections

e Defined as from the start of the test procedure to reading the result

f There is minimal data to inform the pricing, however it is acknowledged that for widespread distribution the costs may need to be lower

### A roadmap for JE diagnostics

Based on the discussion and feedback during the TPP development process, several key discussion points were used to map out considerations both for test development and implementation that can form the basis of a JE diagnostic roadmap (Table 2). The most important output from the discussion was the need for a JE RDT. For this, it was suggested by experts to explore a two-tiered algorithmic approach for JE. This could include testing with a JE RDT, potentially a pan-flavivirus RDT, available at any level of the health system with a confirmatory reference pan-flavivirus specific assay available ideally locally or as an alternative, sub-nationally. JE diagnostic tests need to be incorporated into a diagnostic strategy for all relevant flaviviruses, taking into account changing epidemiology and geographic variation. Patients could be diagnosed in real-time such that a diagnosis is actionable. There could be data-linkage of results, such that the results are informing surveillance, and potentially enabling an impact on outbreak management. Pathogen-based (RNA and/or NS1) methods could also be available, as a suggested 10–20% of JE cases would be expected to be positive with these methods, providing a method of detection for patients with immunosuppression who may not mount a robust antibody response, and also crucial information about molecular epidemiology relevant for vaccine escape [16,41]. Finally, a rapid result would enable consideration of treatment trials.

**Table 2:** Issues of current JE diagnostics and considerations for development and implementation of new JE diagnostic tests.

Discussion point	Considerations for:	
	Product Development	Test implementation
Non-specific presentation of acute encephalitis syndrome (AES)	Simple to use and cost-saving design	Low-cost tests accessible to a large population at low levels of the health system to allow for widespread use
Transient viraemia and rapid IgM-to-IgG switching or absent IgM response in secondary flavivirus infections [42]	Need to consider incorporating alternative analytes such as IgG and host biomarkers	Utilisation of a multi-tiered testing algorithm for initial and confirmatory testing
Serological cross-reactivity of co-circulating flaviviruses and from prior JEV vaccination	Provision of a variety of patient samples of relevant circulating flaviviruses and vaccinees to developers to allow fine tuning of assays in development, realistic clinically indistinguishable condition testing during test development, not freezer studies of confirmed JE versus healthy	Need for more specific tests that allow confident assignment of the causative agent
Only severe cases are diagnosed	Product developers need access to a range of patient samples from different severities of illness, including presenting as an undifferentiated febrile illness (see point above)	Test roll-out in decentralised locations to encourage testing of patients early in disease, before AES symptoms develop
Sample constraints: cerebrospinal fluid (CSF) offers higher specificity but is invasive and often unavailable. Difficulty performing LP and obtaining CSF	Assess diagnostic accuracy across variety of specimen types with blood as the primary focus	Accepting lower sensitivity/specificity of RDTs used with blood and roll-out of any new tests as part of a multi-tiered algorithm with escalating sampling complexity. Investigate and address reasons for not performing LP for tests that require CSF.
Lack of laboratory capacity to perform testing at Level 2 healthcare settings (district hospitals)	Emphasise simple and cost-saving test design	Need for tests that are low-cost and accessible also in low level health facilities and that could be used as a screening test as part of a testing algorithm. Improve laboratory infrastructure alongside new tests that are being developed
Limitations in the sensitivity and specificity of available diagnostics	Any new test needs to outperform existing IgM/IgG based tests	Implementation of a multi-tiered testing algorithm which utilises RDTs as screening tests followed by increasingly more specific and complex tests in reference/central laboratories (level 1/2 healthcare settings) for confirmatory testing.
Technical limitations: PRNT and other seroneutralisation assays are limited by cost, complexity and turnaround time, and are also challenging to interpret in endemic areas	Need to develop replacements for PRNT. Novel methods such as Luminex assays are being developed to replace existing viral seroneutralisation methods [43–47], and these could potentially be performed on blood eluted from filter paper [48].	
Surveillance gaps: non-uniform reporting and evolving encephalitis epidemiology	Connectivity and digital link should be considered during test development to allow transfer of test results into the local surveillance system (e.g., via a connected reader)	Utilisation and recording protocols for data generated by any diagnostic tool at any level to inform the local public health program

For example, such an algorithm could have the following components, if the RDT is JE positive or equivocal:

1. The RDT and additional samples will be sent to a Reference Laboratory (level 1/2 healthcare settings) for confirmatory testing. Confirmatory testing will not be restricted to highly specialised virology laboratories, incorporating methods that are accessible and timely.
2. The patient is referred for specialist JE care with access to an intensive care unit, ideally a neuro-intensive care unit
3. If possible, the patient samples will be tested by reverse transcription polymerase chain reaction (RT-PCR)
4. If possible, the patient will be started on a specific treatment, or enrolled in a JE treatment trial

A patient with an RDT that is equivocal or negative should trigger investigation for alternative diagnoses using all other available tests.

Novel diagnostic test development is not insurmountable. There is a need to work with industry developers and policy makers to collaborate on a technically suitable test that can be implemented within an amenable health system. Although the expert feedback demonstrated the need for improved tests, many questions regarding JE diagnostic testing remain and additional research into the virus, its epidemiology and pathophysiology need to continue (Table 3).

**Table 3:** Future research.

JEV infection presenting as an undifferentiated febrile illness	There are minimal data on JEV infection presenting as an undifferentiated febrile illness [27,49]. It is not clear what proportion of patients have detectable JEV RNA in body fluids, or the associated morbidity and mortality. This may be an important aspect to capture in future diagnostics, to help institute early antiviral or immunomodulatory treatment.
JE treatment	There have been a handful of clinical trials evaluating specific JE treatment, antivirals (ribavirin and interferon alpha 2a) and intravenous immunoglobulins [4]. None have demonstrated improved outcome in JE, though arguably they were underpowered [4]. Keeping in mind the advances that have been made in antiviral treatment since COVID-19 pandemic, this area demands further work.
Epidemiology of neurotropic flaviviruses	There are issues with the accuracy and accessibility of existing JE diagnostics [16]. JE case reporting is patchy and frequently based on serum testing. Knowledge of the true disease burden of JE alongside other endemic neurotropic flaviviruses is limited.
Molecular epidemiology of JEV	JEV RNA is rarely detected in human samples, and we have very limited understanding of the molecular epidemiology [50–52]. This relates to the next point on vaccine failure, e.g., vaccine escape.
Vaccines	There is a need for more data on vaccine immunogenicity and the need for booster doses. Current vaccines are based on JEV genotype 3, however the predominant JEV genotype is now genotype 1, though this may not be the case in human isolations, where there are few data. There are suggestions that current vaccines may be less effective against genotype 1 [53]. There are uncharacterised issues with the vaccine supply chain, vaccine uptake, and potential substandard and falsified vaccines.

## Discussion

This is the first attempt to systematically understand and collate the views of interdisciplinary stakeholders on the need for updated JE diagnostics and map out a roadmap for their use. There has been a stagnation in the field of JE diagnostics, with suggestions that the battle has been won with vaccination. However, there is evidence of expansion of JEV into new areas, and ongoing cases in areas that are vaccinating [9]. There are poorly articulated or characterised issues relating to vaccine effectiveness, vaccine quality, and vaccine uptake. Furthermore, existing diagnostics are not sufficiently accurate or timely to significantly impact patient management. The Delphi process enabled structured, iterative and anonymous feedback from a wide range of experts. The one-day meeting was carefully planned to encourage participation, utilising different modes of communication, with opportunities for comments, breakout groups and electronic voting. We used a validated systematic approach to consensus building, with transparency in developing and refining characteristics. The meeting and discussions facilitated resolution of disagreement, with expertise in a wide range of fields. A hybrid meeting facilitated participation from a wide geographical area, allowed region-specific perspectives, and ensured alignment with user needs and feasibility depending on country infrastructure.

There was considerable debate over whether a new JE test is needed primarily for surveillance or for patient management. This is not an uncommon issue when focussing diagnostic priorities for vaccine preventable diseases [54,55]. There was strong advocacy from participants that the priority should be a test for patient management; therefore, we focused on a test for patient management, with the knowledge that such a test will also inform surveillance. Another noteworthy topic of discussion was the clinical accuracy of a diagnostic test, with participants voicing concerns about the metric of a test; raising the bar too high would not be feasible, or conversely, if the bar was too low (for example, a specified minimal criteria < 90% sensitivity and <90% specificity) then results would not be used. Ultimately, there was agreement on the minimal clinical accuracy metrics, and nearly consensus on the preferred characteristics.

We acknowledge that the study did not include a formal sample size calculation as we relied on pragmatic sampling, although we did attempt as much as possible for this to be systematic, inclusive and diverse. Representatives from nine of twenty-five endemic countries did not contribute to this work, although we attempted to include all. Similarly, not all participants contributed to all the surveys or attended the meeting. In the survey, a lack of responses may have led to attrition bias. It may have been beneficial for all participants to attend the meeting in-person as the hybrid meeting may have limited contribution from those online, however it did not seem appropriate for environmental sustainability to use air travel for a one-day meeting. There may have been group dynamics influencing the discussion, and potentially limited time for discussion.

There was considerable discussion regarding the parameters of sensitivity and specificity, and in the end a lack of agreement regarding the preferred specificity. Many participants reported that the specificity needed to be very high (>95%) to ensure that it was clinically applicable, for end-users to be confident in a positive test result, stopping unnecessary antibiotics and referring for supportive care. However, other participants reported that such a high threshold would set the bar too high for product developers. Either way, these are suggested parameters with no absolute thresholds. There is a unique consideration in the field of flavivirus diagnostics which is not present for many other infection diagnostics, and that is the almost inevitable presence of other viruses that are abundant, can cause similar syndromes in many cases, and give considerable overlap in the results of diagnostics based on measuring antibody responses. This means that any flavivirus antibody diagnostic test needs to be able to differentiate between flaviviruses in order to achieve sufficient specificity. It should be stressed that the primary motivation for a multiplex test arises from the need to address the challenge of improving specificity, although diagnosing other heterologous flaviviruses is arguably an additional benefit for the same reasons that we need to diagnose JE.

The optimal sample matrices were discussed, more from the perspective of what is feasible, than those that offer maximal sensitivity. It is clear that reliance on lumbar puncture for diagnosis is not always practical, and there is a need for blood-based testing. We acknowledge that whole blood may be more sensitive for PCR testing [56], and that there is literature suggesting alternative analytes such as IgA [57,58].

This TPP development process highlighted research questions that remain unaddressed, and additional work needs to be done to help contribute to new JE diagnostic tests within existing or evolving algorithms for suspected neurological infections. We do not understand how frequently JEV infection presents as an undifferentiated febrile illness, and what proportion of such cases go on to develop neurological infection. We do not have a specific treatment for JE. We have a poor understanding of the molecular epidemiology of JEV, and of the epidemiology of other neurotropic flaviviruses, particularly when and where there are novel sporadic cases. We do not understand whether the vaccine is working, and if the vaccine is not working, why. To help start addressing these concerns, we need to develop and evaluate new JE diagnostics that address the TPP characteristics. The TPP is accompanied by a suggested roadmap for JE diagnostics development and implementation in the context of other endemic flaviviruses.

In the final TPP, the minimal criteria set out a pragmatic and realistic set of characteristics for a novel JE diagnostic. Many of the minimal criteria are already achieved by existing JE IgM ELISA methods. Outstanding minimal criteria that are not achieved by the JE ELISA and are perceived as a priority are: 1) a test that impacts on patient management, 2) minimal cross-reactivity, 3) clinical accuracy metrics of ≥90% sensitivity and ≥92% specificity. Overall, there is a clear desire for a RDT to be performed together with a more accurate confirmatory assay. This will require both researchers, industry developers and policy makers to work together to develop a test that can be placed within the existing and developing infrastructures of health systems. These plans are in line with the WHO Encephalitis Technical Briefing and the WHO Arbovirus Initiative [15,59].

## Supporting information

S1 Appendix Survey 1: First round of the Delphi survey sent to participants. (PDF)

S2 Appendix Survey 2: Second round of the Delphi survey sent to participants. (PDF)
