# Supplementary material for: A target product profile and roadmap for Japanese encephalitis diagnostics in endemic areas in the context of other flaviviruses
Source: PLoS Negl Trop Dis. 2026 Sep 8;20(9):e0014683. doi: 10.1371/journal.pntd.0014683 (PMC13581209; doi:10.1371/journal.pntd.0014683)
Supplement: Supplementary file 1 [file pntd.0014683.s001.pdf]

https://forms.cloud.microsoft/Pages/ResponsePage.aspx?id=B3jtTq3rWkGnqZFwlH9OrtEpk1XLfjdPlw88XRaVD8FUNzNMSlpJMEdZTk1aWkU5TEFEUkZLMk5LSy4u

Development of a Target Product Profile for Japanese encephalitis diagnostics in Asia

What is a target product profile (TPP)? A TPP is a planning tool for the development of health products. TPPs specify the intended use, target populations and desired attributes of products and guide product development programmes. In the broader context of public health, TPPs have emerged as tools to set research and development (R&D) targets and priorities for funders and developers. (https://www.who.int/observatories/global-observatory-on-health-research-and-development/analyses-and-syntheses/target-product-profile/who-target-product-profiles)

What is the process for TPP development? We started with online interviews with a handful of stakeholders (clinicians and scientists) across Asia. This identified a number of key points requiring further input, that we have incorporated into the following online survey. This survey is being sent out to a larger number of participants, identified from track-record and publications in the field. It is termed a 'Delphi survey', as we will receive feedback, amend the survey and send it out again until we have reasonable consensus. This will be discussed further during a hybrid meeting on the 8th October 2025, during which characteristics of the TPP will be finalised. Those who respond to the survey and provide contact details where requested will be invited to this hybrid meeting.

What is the aim of this TPP? Japanese encephalitis (JE) remains a leading identified cause of neurological infection in Asia and a neglected disease. Issues with current diagnostics remain a barrier to instituting appropriate management and public health measures. We aim to develop a TPP for JE diagnostics in the context of other neurotropic flaviviruses in Asia, to better understand what improvements are needed.

Consent and data governance Data submitted by survey participants will be anonymised and stored securely. Participation is voluntary, and participants are free to opt out of taking part in this research at any time (including following completion and submission of the survey). Submission of this completed survey is taken as consent to participate in the research.

Survey Format

- Each survey question below begins with a table containing a 'minimal' and a 'preferred' column.
- A 'minimal' characteristic refers to the lowest acceptable standards for a diagnostic test, while a 'preferred' characteristic refers to the ideal or optimal features of a diagnostic test.
- Below each table there are 2 questions structured as agree/disagree ratings, with 5 options corresponding to the level of agreement (ranging from 'strongly disagree' to 'strongly agree').
- For each survey question, please answer both minimal and preferred.
- Finally, for each survey question, please provide further details/rationale to your answer where requested.
- The survey ends with questions 15-18, aimed at gathering anonymous details of respondents' work in the field, and question 19, asking for contact details (optional).

We greatly appreciate your time and input into this important topic.

When you submit this form, it will not automatically collect your details like name and email address unless you provide it yourself.

* Required

1

What should be the intended use/intended purpose of a JE diagnostic test?

*

MINIMAL	PREFERRED
Diagnosis of acute symptomatic JEV infection for surveillance	Diagnosis of acute symptomatic JEV infection for patient management and surveillance

	Strongly disagree	Disagree	Neutral	Agree	Strongly agree
Minimal	◯	◯	◯	◯	◯
Preferred	◯	◯	◯	◯	◯

2

Please provide any further details/rationale to your answer regarding intended use/purpose of a JE diagnostic test. *

Enter your answer

3

Who should be the intended users of a JE diagnostic test, and in what setting would it be used?

*

MINIMAL	PREFERRED
Reference laboratory	Any healthcare facility including primary care, no need for specialist laboratory facilities

	Strongly disagree	Disagree	Neutral	Agree	Strongly agree
Minimal	◯	◯	◯	◯	◯
Preferred	◯	◯	◯	◯	◯

4

Please provide any further details/rationale to your answer regarding the intended user and use setting for a JE diagnostic test. *

Enter your answer

5

What would you consider to be the minimal acceptable sensitivity and specificity values for a JE diagnostic test, and what would be the preferred sensitivity and specificity values?

*

CHARACTERISTIC	MINIMAL	PREFERRED
Clinical sensitivity	>95%	>97%
Clinical specificity	>97%	>99%

	Strongly disagree	Disagree	Neutral	Agree	Strongly agree
Minimal	○	○	○	○	○
Preferred	○	○	○	○	○

6

Please provide any further details/rationale to your answer regarding the clinical performance of a JE diagnostic test, and whether sensitivity or specificity is more important. *

Enter your answer

7

What is the minimal and preferred analytical performance for a JE diagnostic test?

*

MINIMAL	PREFERRED
Can distinguish from dengue	High specificity against all neurotropic flaviviruses

	Strongly disagree	Disagree	Neutral	Agree	Strongly agree
Minimal	◯	◯	◯	◯	◯
Preferred	◯	◯	◯	◯	◯

8

Please provide any further details/rationale to your answer regarding the analytical performance of a JE diagnostic test. *

Enter your answer

9

What specimen type would be appropriate for a JE diagnostic test?

*

MINIMAL	PREFERRED
Whole blood or CSF	Finger-prick blood samples. Multiple bodily fluids in parallel (e.g. urine, saliva, nasal/throat swabs)

	Strongly disagree	Disagree	Neutral	Agree	Strongly agree
Minimal	◯	◯	◯	◯	◯
Preferred	◯	◯	◯	◯	◯

10

Please provide any further details/rationale to your answer regarding the specimen type for a JE diagnostic test. *

Enter your answer

11

What would be the minimal and preferred turnaround time for a JE diagnostic test? *

MINIMAL	PREFERRED
24-48 hours	15-30 mins

	Strongly disagree	Disagree	Neutral	Agree	Strongly agree
Minimal	○	○	○	○	○
Preferred	○	○	○	○	○

12

Please provide any further details/rationale to your answer regarding turnaround time for a JE diagnostic test. *

Enter your answer

13

How many steps would be acceptable for the assay procedure of a JE diagnostic test?

*

MINIMAL	PREFERRED
Multiple steps involved, for example 3-6	One-step involved

	Strongly disagree	Disagree	Neutral	Agree	Strongly agree
Minimal	◯	◯	◯	◯	◯
Preferred	◯	◯	◯	◯	◯

14

Please provide any further details/rationale to your answer regarding the assay procedure of a JE diagnostic test. *

Enter your answer

15

Please describe your type of expertise related to JEV (e.g. clinical, public health, epidemiology, basic sciences) *

Enter your answer

16

Please specify the number of years of work related to JEV *

Enter your answer

17

Please describe your current role and place of work *

Enter your answer

18

Please specify your location (country) *

Enter your answer

19

Please provide your name and email address if you are happy to do so, and to ensure that we keep you updated. This information will be stored separately from the analysis of the responses to the TPP questions above.

Enter your answer
