# Supplementary material for: A target product profile and roadmap for Japanese encephalitis diagnostics in endemic areas in the context of other flaviviruses
Source: PLoS Negl Trop Dis. 2026 Sep 8;20(9):e0014683. doi: 10.1371/journal.pntd.0014683 (PMC13581209; doi:10.1371/journal.pntd.0014683)
Supplement: Supplementary file 2 [file pntd.0014683.s002.pdf]

Development of a Target Product Profile for Japanese Encephalitis Diagnostics (Revised)

TPP process Thank you very much for completing the previous survey. We have reviewed the responses and revised the TPP accordingly. Please see the updated information marked with bold text. We are now sending it to you again with the aim of achieving reasonable consensus.

Consent and data governance The data you submit via this survey will be anonymised and stored securely. Your participation is voluntary, and you are free to opt out of taking part in this research at any time (including following completion and submission of the survey). Submission of your completed survey is taken as consent to participate in the research.

Survey Format

- Based on your responses to the first survey, there are now two sections, one for a JE diagnostic for patient management and the other for a JE diagnostic for surveillance.
- Each survey question below begins with a table containing a 'minimal' and a 'preferred' column.
- A 'minimal' characteristic refers to the lowest acceptable standards for a diagnostic test, while a 'preferred' characteristic refers to the ideal or optimal features of a diagnostic test.
- Below each table there are 2 questions structured as agree/disagree ratings, with 5 options corresponding to the level of agreement (ranging from 'strongly disagree' to 'strongly agree').
- For each survey question, please answer both minimal and preferred.
- Finally, for each survey question, please provide further details/rationale to your answer where requested.

We greatly appreciate your time and input into this important topic.

* Required

1

What should be the intended use/purpose of a JE diagnostic test? *

MINIMAL	PREFERRED
**Diagnosis of symptomatic JEV infection for surveillance to improve public health**	**Diagnosis of symptomatic JEV infection for acute care and surveillance to improve patient management and public health**

	Strongly Disagree	Disagree	Neutral	Agree	Strongly Agree
Minimal	◯	◯	◯	◯	◯
Preferred	◯	◯	◯	◯	◯

2

Please provide any further details/rationale to your answer regarding intended use/purpose of a JE diagnostic test. *

The following section is related to a JE diagnostic tool used for surveillance purposes.

3

In what setting should a new JE diagnostic test be used to be most useful for surveillance purposes? (https://shorturl.at/h6nCL and image below for reference)

*

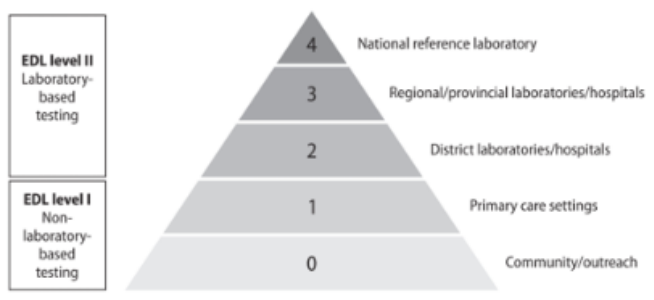

EDL: WHO model list of essential in vitro diagnostics; IVDs: in vitro diagnostics.
Source: adapted from (1).

MINIMAL	PREFERRED
**Regional/provincial laboratories/hospitals or above (≥ level 3)**	**District laboratories/hospitals or above (≥ level 2)**

	Strongly disagree	Disagree	Neutral	Agree	Strongly agree
Minimal	◯	◯	◯	◯	◯
Preferred	◯	◯	◯	◯	◯

4

Please provide any further details/rationale to your answer regarding the intended use setting for a JE diagnostic test for surveillance purposes. *

5

What would you consider to be the minimal and preferred sensitivity and specificity values for a JE diagnostic test for surveillance purposes?
*tested against other endemic neurotropic flaviviruses, e.g. dengue virus, Zika virus ± West Nile virus.

*

CHARACTERISTIC	MINIMAL	PREFERRED
Clinical sensitivity	**≥90%**	**≥95%**
Clinical specificity	**≥90%**	**≥97%**

	Strongly disagree	Disagree	Neutral	Agree	Strongly agree
Minimal	◯	◯	◯	◯	◯
Preferred	◯	◯	◯	◯	◯

6

Please provide any further details/rationale to your answer regarding the clinical performance of a JE diagnostic test for surveillance purposes. *

7

What is the minimal and preferred multiplexing capacity for a JE diagnostic test for surveillance purposes? *

MINIMAL	PREFERRED
**No - Single test for JEV**	**Yes - Simultaneous detection of JE and other flavivirus**

	Strongly Disagree	Disagree	Neutral	Agree	Strongly Agree
Minimal	◯	◯	◯	◯	◯
Preferred	◯	◯	◯	◯	◯

8

Please provide any further details/rationale to your answer regarding the multiplexing capacity of a JE diagnostic test for surveillance purposes. *

9

What specimen type would be appropriate for a JE diagnostic test for surveillance purposes?

*

MINIMAL	PREFERRED
**Blood (serum, plasma or whole blood) or CSF**	**Finger-prick blood samples.**

	Strongly disagree	Disagree	Neutral	Agree	Strongly agree
Minimal	◯	◯	◯	◯	◯
Preferred	◯	◯	◯	◯	◯

10

Please provide any further details/rationale to your answer regarding the specimen type for a JE diagnostic test for surveillance purposes. *

11

What would be the minimal and preferred duration for the test procedure for surveillance purposes? *i.e. starting the test procedure to reading the result, not from sample submission to the laboratory to reporting the result.* *

MINIMAL	PREFERRED
**≤8hrs**	15-30 mins

	Strongly disagree	Disagree	Neutral	Agree	Strongly agree
Minimal	◯	◯	◯	◯	◯
Preferred	◯	◯	◯	◯	◯

12

Please provide any further details/rationale to your answer regarding duration of the test procedure for a JE diagnostic test for surveillance purposes. *

13

How many timed steps would be acceptable for the assay procedure of a JE diagnostic test for surveillance purposes? (A timed step being a process that involves an incubation period).

*

MINIMAL	PREFERRED
**Multiple(≥2) steps involving waiting for a fixed time period**	**One step involving waiting for a fixed time period**

	Strongly disagree	Disagree	Neutral	Agree	Strongly agree
Minimal	◯	◯	◯	◯	◯
Preferred	◯	◯	◯	◯	◯

14

Please provide any further details/rationale to your answer regarding the assay procedure of a JE diagnostic test for surveillance purposes. *

The following section is related to a JE diagnostic tool for patient management purposes.

15

In what setting should a new JE diagnostic test be used to be most useful for patient management purposes? (https://shorturl.at/h6nCL and image below for reference)

*

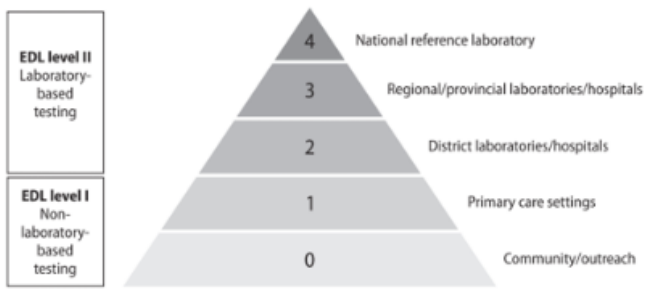

EDL: WHO model list of essential in vitro diagnostics; IVDs: in vitro diagnostics.
Source: adapted from (1).

MINIMAL	PREFERRED
**Regional/provincial laboratories/hospitals or above (≥ level 3)**	**District laboratories/hospitals or above (≥ level 2)**

	Strongly disagree	Disagree	Neutral	Agree	Strongly agree
Minimal	◯	◯	◯	◯	◯
Preferred	◯	◯	◯	◯	◯

16

Please provide any further details/rationale to your answer regarding the intended use setting for a JE diagnostic test for patient management purposes. *

17

What would you consider to be the minimal and preferred sensitivity and specificity values for a JE diagnostic test for patient management purposes? *tested against other endemic neurotropic flaviviruses, e.g. dengue virus, Zika virus ± West Nile virus.

*

CHARACTERISTIC	MINIMAL	PREFERRED
Clinical sensitivity	**≥90%**	**≥95%**
Clinical specificity	**≥90%**	**≥97%**

	Strongly disagree	Disagree	Neutral	Agree	Strongly agree
Minimal	◯	◯	◯	◯	◯
Preferred	◯	◯	◯	◯	◯

18

Please provide any further details/rationale to your answer regarding the clinical performance of a JE diagnostic test for patient management purposes. *

19

What is the minimal and preferred multiplexing capacity for a JE diagnostic test for patient management purposes? *

MINIMAL	PREFERRED
**No - Single test for JEV**	**Yes - Simultaneous detection of JE and other flavivirus**

	Strongly Disagree	Disagree	Neutral	Agree	Strongly Agree
Minimal	◯	◯	◯	◯	◯
Preferred	◯	◯	◯	◯	◯

20

Please provide any further details/rationale to your answer regarding the multiplexing capacity of a JE diagnostic test for patient management purposes. *

21

What specimen type would be appropriate for a JE diagnostic test for patient management purposes?

*

MINIMAL	PREFERRED
**Blood (serum, plasma or whole blood) or CSF**	**Finger-prick blood samples.**

	Strongly disagree	Disagree	Neutral	Agree	Strongly agree
Minimal	◯	◯	◯	◯	◯
Preferred	◯	◯	◯	◯	◯

22

Please provide any further details/rationale to your answer regarding the specimen type for a JE diagnostic test for patient management purposes. *

23

What would be the minimal and preferred duration for the test procedure for patient management purposes? *i.e. starting the test procedure to reading the result, not from sample submission to the laboratory to reporting the result.* *

MINIMAL	PREFERRED
**≤8hrs**	15-30 mins

	Strongly disagree	Disagree	Neutral	Agree	Strongly agree
Minimal	◯	◯	◯	◯	◯
Preferred	◯	◯	◯	◯	◯

24

Please provide any further details/rationale to your answer regarding duration of the test procedure for a JE diagnostic test for patient management purposes. *

25

How many timed steps would be acceptable for the assay procedure of a JE diagnostic test for patient management purposes? (A timed step being a process that involves an incubation period).

*

MINIMAL	PREFERRED
**Multiple(≥2) steps involving waiting for a fixed time period**	**One step involving waiting for a fixed time period**

	Strongly disagree	Disagree	Neutral	Agree	Strongly agree
Minimal	◯	◯	◯	◯	◯
Preferred	◯	◯	◯	◯	◯

26

Please provide any further details/rationale to your answer regarding the assay procedure of a JE diagnostic test for patient management purposes. *

27

Please briefly describe what JE diagnostic(s) are currently available to you and the duration of time from sample submission to the laboratory to the result being reported in your setting.

28

Please provide your name and email address if you are happy to do so, and to ensure that we keep you updated. This information will be stored separately from the analysis of the responses to the TPP questions above.

This content is neither created nor endorsed by Microsoft. The data you submit will be sent to the form owner.

Microsoft Forms
