# Supplementary material for: A target product profile and roadmap for Japanese encephalitis diagnostics in endemic areas in the context of other flaviviruses
Source: PLoS Negl Trop Dis. 2026 Sep 8;20(9):e0014683. doi: 10.1371/journal.pntd.0014683 (PMC13581209; doi:10.1371/journal.pntd.0014683)
Supplement: Supplementary file 3 [file pntd.0014683.s004.docx]

July 2026

Dear Editorial team and reviewers,

Re:

PNTD-D-26-00564
Full title: A target product profile and roadmap for Japanese encephalitis diagnostics in endemic areas, in the context of other flaviviruses
PLOS Neglected Tropical Diseases

We are grateful for your detailed review of our manuscript, and useful suggestions. We provide a point-by-point response below in dark blue.

Kind regards,

Dr Tehmina Bharucha, on behalf of all the authors.

MRCP FRCPath DTM&H DPhil

Consultant in Infectious Diseases and Microbiology, Imperial College Healthcare NHS Trust, London, UK

Postdoctoral Clinical Fellow, The Francis Crick Institute, London, UK

[t.bharucha@doctors.org.uk](mailto:t.bharucha@doctors.org.uk)

**Reviewers' Comments:**

Reviewer's Responses to Questions

**Key Review Criteria Required for Acceptance?**


As you describe the new analyses required for acceptance, please consider the following:


**Methods**


-Are the objectives of the study clearly articulated with a clear testable hypothesis stated?

-Is the study design appropriate to address the stated objectives?

-Is the population clearly described and appropriate for the hypothesis being tested?

-Is the sample size sufficient to ensure adequate power to address the hypothesis being tested?

-Were correct statistical analysis used to support conclusions?

-Are there concerns about ethical or regulatory requirements being met?

Reviewer #1: Delphi methodology and consensus analyses were appropriately conducted and presented. Study participants were representative of JEV endemic areas and all attempts made to be inclusive of all areas impacted by JEV infection. Limitations to participant engagement was described appropriately.

We are pleased that you are satisfied with the methodology.

Reviewer #2: The study objectives are articulated with a feasible hypothesis.
The study design looks appropriate to prepare a regional target product profile for Japanese Encephalitis. However, a clear organization is needed in the Methods Section as the Methods are also described in the Results section. A flowchart of the TPP process should be included in the Methods section.

Thank you for the useful suggestion. We have created a Figure illustrating the TPP process, Fig 1 Overview of the TPP process. We have also removed the first paragraph of the Results section, the Overview of the TPP process, and inserted pertinent information on the findings into other paragraphs.

The first version of the JE TPP was prepared based on the interviews of 5 experts. A clear justification for the limited number of experts should be included in the manuscript.

The manuscript has been updated to add clarity with regard to the experts invited for online interviews. In the Methods section, second paragraph “TPP process”, first sentence, page 10, “A representative sample of participants from diverse disciplines and countries was invited to participate in initial scoping online interviews (which were conducted on Zoom), with the aim of identifying common themes relating to issues with JE diagnostics. The interviews were performed in series until data saturation was achieved.”

Further clarification and justification for the number of participants in the 1st survey and 2nd survey is also needed. The study lacks of statistical analysis that can support observations.

All participants identified from the searches were invited to participate in the online surveys, and this was deemed a pragmatic sample size. In the Discussion section, fourth paragraph, first sentence, page 37, we have added a sentence to acknowledge this limitation: “We acknowledge that the study did not include a formal sample size calculation as we relied on pragmatic sampling, although we did attempt as much as possible for this to be systematic, inclusive and diverse.”

No ethical concerns.

**Results**


-Does the analysis presented match the analysis plan?

-Are the results clearly and completely presented?

-Are the figures (Tables, Images) of sufficient quality for clarity?

Reviewer #1: Some consideration on rank order of test component criteria should be made (i.e. which of the testing criteria would be critical and which would be beneficial but would not preclude further development and/or evaluation if the minimal standards highlighted in the TPP were not achieved.

The TPP separates minimal and preferred criteria for a future test. The minimal criteria have been agreed as being achievable. Further, the existing JE ELISA methods fulfil many of the minimal criteria. The outstanding minimal criteria that are not achieved by the JE ELISA are: 1) a test that impacts on patient management, 2) minimal cross-reactivity, 3) clinical accuracy metrics of ≥90% sensitivity and ≥92% specificity. We have added text in the Discussion to highlight this: “In the final TPP, the minimal criteria set out a pragmatic and realistic set of characteristics for a novel JE diagnostic. Many of the minimal criteria are already achieved by existing JE IgM ELISA methods. Outstanding minimal criteria that are not achieved by the JE ELISA and are perceived as a priority are: 1) a test that impacts on patient management, 2) minimal cross-reactivity, 3) clinical accuracy metrics of ≥90% sensitivity and ≥92% specificity.”

The role of immunomodulatory therapies on diagnostic testing performance and applicability of testing modality should be incorporated in the TPP. The use of B cell depletion therapies for a litany of clinical ailments including multiple sclerosis, rheumatoid arthritis, lymphoma etc. can negate the utility of serological-based diagnostic testing, requiring the use of antigen or nucleic acid-based testing. Although more commonly used in areas of the world not typically impacted by JEV, use of these therapies does occur in JEV endemic areas and likely will be increasing over time. A caveat to this could be included in the tiered testing discussion that was described from addressing heterologous flavivirus circulation but could also incorporate the use of different targets based on immunomodulatory treatment, immunosuppressed patients and could be a strong consideration based on the timing post-symptom onset (i.e. highly acute samples presented with acute febrile symptomology prior to inset of neurological symptoms. Furthermore, the use of nucleic acid-based testing should be considered in the context of whole blood as a sample testing matric for which a series of flaviviruses have demonstrated efficient detection of viral RNA over extended time periods.

Thank you for pointing out these important considerations. We have incorporated this through the manuscript in different sections:

- In the Introduction, we have edited the following sentence on page 8: “The pathogen, referring primarily to JEV nucleic acid, is rarely detected in human body fluids, rendering common testing methods, such as molecular diagnosis of JEV nucleic acid, less useful.”
- In the Results, in Table 1, analysis type, Notes from discussion/rationale, we have added the following sentence: “It is acknowledged that, although pathogen-based methods such as nucleic acid and protein detection, do not demonstrate high diagnostic yield, there is an important role for them. Aside from improved specificity and potential for tracking mutations and variants, these methods have a role in diagnosing the increasing number of patients with immunosuppression for whom antibody-based methods may not be useful.”
- In the Results, in the section on “A roadmap for JE diagnostics”, a sentence has been edited: “Pathogen-based (RNA and/or NS1) methods could also be available, as a suggested 10-20% of JE cases would be expected to be positive with these methods, providing a method of detection for patients with immunosuppression who may not mount a robust antibody response, and also crucial information about molecular epidemiology relevant for vaccine escape (16, 41)”.

The concept of improved serological specificity through IgG depletion is not addressed in the future considerations section. Given its potential importance as part of the two-tiered testing description for addressing specific flavivirus diagnoses, I would suggest the be highlighted in the future assay considerations section.

We have referenced IgG depletion in the introduction (page 9), citing work that has already been performed to demonstrate its utility: “IgM-VNT, involving immunoglobulin G (IgG) depletion of sera prior to VNT, has been utilised to improve interpretation and may be performed in a single serum sample (20). “

Reviewer #2: The results presented require a clear description. For example, the criteria for concordance, feedback ratios, the number of participants (“some felt”), and criteria for too low sensitivity/specificity should be included in the text.
The Consensus building on TPP characteristics’ lack ratios or % in the text. Percentage of comments out of 44 experts that refer to the use of a test of patient management and POC is missing.

Thank you for highlighting these points. We have edited the text to improve the clarity of the description of the results.

- Agreement and concordance for the scoping interviews has now been detailed.
- In the paragraphs reporting the first and second surveys, the number of participants has been reported.
- For all the results, the percentage agreement is reported in the text.
- The term ‘felt’ has been replaced by ‘reported’.
- The criteria for insufficient sensitivity and specificity have been included: “Another noteworthy topic of discussion was the clinical accuracy of a diagnostic test, with participants voicing concerns about the metric of a test; raising the bar too high would not be feasible, or conversely, if the bar was too low (as specified in the minimal criteria, <90% sensitivity and <90% specificity) then results would not be used.”
- The percentage of participants making free-text comments has been reported.

The results are not clearly presented, as certain methods (overview of the process) and redundant information are included in the Results sections.

The first paragraph on the overview of the process has been removed from the Results section.

Figures have enough quality for clarity.

Flowcharts for the JE diagnostic should be included in the text.

A flowchart providing an overview of the TPP process has been created, see new Fig 1.

Citation of the figures in the text (E.g. Fig 1), and supplementary figures should follow PLOS guidelines.

The citations to figures and supplementary data have been corrected in line with PLOS guidelines.

**Conclusions**


-Are the conclusions supported by the data presented?

-Are the limitations of analysis clearly described?

-Do the authors discuss how these data can be helpful to advance our understanding of the topic under study?

-Is public health relevance addressed?

Reviewer #1: Conclusions are supported by the data presented. The authors interpret findings with caution and indicate complexity appropriately. Some clarification on how clinical presentation of neurological syndromes from heterologous flaviviruses might be impacted by lack of a specific diagnosis given the largely supportive care and lack of treatment options. Heterologous flaviviruses that could also elicit neurological symptoms could included West Nile/Kunjin , Kyasanur Forest, tick-borne encephalitis, Murray Valley encephalitis and Zika viruses.

A sentence has been amended in the discussion, page 38, to expand on this: “It should be stressed that the primary motivation for a multiplex test arises from the need to address the challenge of improving specificity, although diagnosing other heterologous flaviviruses is arguably an additional benefit for the same reasons that we need to diagnose JE.”

The discussion points should be made in the context of what sample matrices would be optimal for disease presentation differences and time post symptom onset. There is no mention of the potential diagnostic utility of IgA testing

A paragraph has been added to the discussion to reflect this: “The optimal sample matrices were discussed, more from the perspective of what is feasible, than those that offer maximal sensitivity. It is clear that reliance on lumbar puncture for diagnosis is not always practical, and there is a need for blood-based testing. We acknowledge that whole blood is more sensitive for PCR testing (56), and that there is literature suggesting alternative analytes such as IgA (57, 58).”

Reviewer #2: Conclusions are partially supported by the data presented. Data results must be clearly presented and organized in the manuscript.

Detailed above, we have edited the results section to make the presentation of the results clearer.

The study mentioned current issues with the JE diagnostic and the consideration of new diagnostic tests.
The authors discussed how the TPP for JE has highlighted several topics for JEV that require further research and attention.
Public health relevance of the Target product profile for Japanese encephalitis is addressed.

**Editorial and Data Presentation Modifications?**


Use this section for editorial suggestions as well as relatively minor modifications of existing data that would enhance clarity. If the only modifications needed are minor and/or editorial, you may wish to recommend “Minor Revision” or “Accept”.

Reviewer #1: The authors use the term “point-of-care test”; however, a descriptor of “rapid diagnostic test” would be a more appropriate term that would encapsulate the different attributes highlighted in the target product profile given that some of the benefits would be surveillance-oriented as well as direct care might not be available and assessment of alternative etiologies would be sought or patients would be either enrolled in JEV clinical treatment trials or admission to neuro-intensive care unit more efficiently.

The term ‘Point-of-care-test’ has been replaced by ‘rapid diagnostic test’ throughout the manuscript.

Introduction line 135: some mention of whether Culex control is utilized as a public health measure in response to documented human clinical disease cases.

A sentence has been added to mention the role of Culex control in the last sentence of the third paragraph of the introduction (page 7): “In the context of outbreaks, JE confirmation may also lead to mosquito-control measures, such as intermittent irrigation and space spraying of insecticides (1).”

Line 230-Overiew of the Process: should this description be included in the methods section as opposed top the description of the findings?

The first paragraph of the Results, the Overview of the Process, has been removed.

Table 1: Test procedures- the topic of multiplexing for the assessment (i.e. rule-out) of heterologous circulating flaviviruses is described in the context of two-tiered testing. Some description of the relative impact that multiplex testing can have on assay performance standards should be included (i.e. principally, the negative impact on test sensitivity).

We agree that if the format involves converting multiple assays into a single reaction with the same sample volume there is the potential to reduce sensitivity. We have added this point to the text, in table 1, in the section on the test procedure, in multiplexing, in the Notes from the discussion/ rationale (page 22): “While multiplex testing is attractive, it is also associated with increased costs and, if reduced to a single assay there is the potential for reduced sensitivity.”

Table 1: Ease of Use- a determination of a minimal standard for four steps should be presented in the context of non-inferiority of current diagnostics assays in use.

The context for the minimal standard has been added in table 1, in the section on ease of use, in the notes from discussion/rationale (page 24): “This is modelled on existing ELISA methods which are the standard diagnostic method in use.”

Table 1: Test kit stability/operational conditions- there is no consideration of how the shipment and storage of testing components could be augmented by the use of desiccants. The impact on nucleic acid testing has been demonstrated to be extended on FTA cards in which desiccants have been utilized.

The prospect of improved stability of analytes by the use of desiccants has been added to the text, in table 1, operational characteristics, test kit stability/ operational conditions, Notes from discussion/ rationale (page 26): “We acknowledge that desiccants can improve the stability of analytes (2).”

Table 1: kit controls- some discussion of sample viability determination should be included (determination of the level of hemolysis, presence of inhibitors etc.) should be considered.

We have added discussion to clarify this: “We would expect that industry standards relevant for specific tests will be fulfilled, for example a control line in any RDT.”

Line 320: a reference for the 10% diagnostic testing rate for NS1/RNA. As described above this would be mandated for immunosuppressed patients.

References have been added (Li *et al* 2012 and Bharucha *et al*, 2020).

Line 331: “RT-PCR”

PCR has been amended to reverse transcription polymerase chain reaction (RT-PCR).

Table 2: given the presentation of product development consideration and test implementation in the table (in that order), the title should mention in the same order.

We agree that this is more logical and have amended the title of Table 2 accordingly: “Issues of current JE diagnostics and considerations for development and implementation of new JE diagnostic tests.”

Reviewer #2: (No Response)

**Summary and General Comments**


Use this section to provide overall comments, discuss strengths/weaknesses of the study, novelty, significance, general execution and scholarship. You may also include additional comments for the author, including concerns about dual publication, research ethics, or publication ethics. If requesting major revision, please articulate the new experiments that are needed.

Reviewer #1: The PLoS Neglected Tropical Diseases Manuscript PNTD-D-26-00564 entitled “A target product profile and roadmap for Japanese encephalitis diagnostics in endemic areas, in the context of other flaviviruses” by Bharucha et al. describes the results of a Delphi process that was utilized to refine the content of target product profile for Japanese encephalitis virus. The investigators identified a consensus on the majority of the components for minimal and ideal performance standards for attributes of the JEV diagnostic test. Emphasis of performance characteristics for which diagnostic testing would be performed as a priority for patient management was relayed by on the overwhelming consensus of participants that this should be the testing priority. The authors should be commended on the thorough addressing a very complicated topic that addresses diagnostic challenges in the context of circulating heterologous flaviviruses that complicates serodiagnosis as well as the historical emphasis on vaccine distribution at the expense of improved JEV diagnostics. General and specific comments for consideration by the authors have been provided.

Reviewer #2: Japanese encephalitis (JE) virus has been one of the most important aetiologic agents of severe neurological infections in Asia and the Pacific regions. The study aimed to develop, for the first time, a regional target product profile (TPP) for JE diagnostics in endemic areas to improve patient management and public health. Initial interviews were performed with five selected experts to prepare a first version of a TPP document. Then, a Delphi methodology was used to obtain consensus from a subgroup of 9.56% (44/460) experts, aiming for≥75% consensus on each JE test characteristic described in the TPP, through two online surveys. Subsequently, a one-day meeting facilitated in-depth discussion with 42 out of 44 participant experts. Overall, seven priority JE characteristics were discussed, and consensus was achieved for all characteristics, with the exception of the preferred JE test sensitivity and specificity (73% agreement). The TPP for the JE test outlines a critical characteristic for future development and implementation in the region. However, the manuscript requires a major revision for further publication consideration.
The abstract requires to integrate and summarize the methods and results of the study. A broad impact of the TPP study should be mentioned as the final sentence.

The abstract has been edited, to include a final sentence on the broad impact of the TPP study: “The final TPP is presented in the paper; this aims to help define the updates needed to better support patient management and public health interventions, to provide a strategic reference document to drive innovation and improve clarity for JE diagnostic test developers.”

Furthermore, the manuscript requires more attention in the methods and results section, including figures and supplementary data. Flowcharts for the TPP process and algorithm should be included in the manuscript. Grammar throughout the text needs to be revised.

The text has been revised accordingly.

Specific comments:
Line 79-99, Revise and integrate the abstract

The abstract has been revised.

Line 111-112, Revise sentence “approximately 15-30% die”

The word “approximately” has been removed.

Line 122, Spell out 16/25

16/25 has been replaced by Sixteen of twenty-five.

Line 143, Add the words “serotypes 1 to 4” in parenthesis

“Dengue viruses has been corrected to “Dengue viruses (serotypes 1-4)”

Line 145-145, Revise sentence “is even lower “

The sentence has been corrected to “Lastly, there appears to be increasing reliance on JEV ELISA testing of serum, for which the specificity is lower when compared to the recommended specimen, CSF (3).”

Line 148, Replace “titer” with “test”

“Titre” has been replaced by “test”.

Line 167, Is “AND diagnos” correct?

Yes, the asterisk was used to incorporate both diagnosis and diagnostics.

Line 186, Suggest including the word” preliminary draft”

The word “preliminary” has been added before “draft”.

Line 190, Describe JE characteristics

The sentence has been revised, replacing the term ‘JE test characteristics’ by ‘themes that emerged from the online scoping interviews’: “Themes that emerged from the online scoping interview were incorporated into the draft TPP and the Delphi survey to obtain feedback from experts on the information needed to support each characteristic.”

Line 217, Provide the name, degree, and affiliation of Dr. Ava Easton

This has been added: “Dr Ava Easton (PhD, Chief Executive of Encephalitis International) …”

Line 271-272, justify the use of the word “too low” in the text

The sentence has been revised to define ‘too low’: “Another noteworthy topic of discussion was the clinical accuracy of a diagnostic test, with participants voicing concerns about the metric of a test; raising the bar too high would not be feasible, or conversely, if the bar was too low (as specified in the minimal criteria, <90% sensitivity and <90% specificity) then results would not be used.”

Line 274, Suggest including the word “revised TPP”

The word draft has been replaced by revised.

Line 283-5, Cite Figures in the text by using the word “Fig.”

All figures are now cited as Fig.

Line 305, Spell out “RDT”

RDT has been replaced by rapid diagnostic test (RDT).

Line 335-338, revise the sentence

The sentence has been revised: “A patient with an RDT that is equivocal or negative will trigger investigation for alternative diagnoses using all other available tests.”

Line 347, revise sentence “we see expansion of JEV into new areas, and ongoing cases in areas that are vaccinating”

The sentence has been revised: “However, there is evidence of expansion of JEV into new areas, and ongoing cases in areas that are vaccinating (9).”

Line 350-351, revise sentence “The scoping interviews reiterated the appetite for this work”

This sentence has been deleted.

Line 373, Spell out “9/25”

9/25 has been replaced by nine of twenty-five.

Table 1. Revise grammar

PLOS authors have the option to publish the peer review history of their article ([what does this mean?](https://track.editorialmanager.com/CL0/https:%2F%2Fjournals.plos.org%2Fplosntds%2Fs%2Feditorial-and-peer-review-process%23loc-peer-review-history/1/010f019eca4de7f5-b991c2de-3b57-4734-bca3-9ff79ba9dd7f-000000/JLIRat9cFF6PDyAQK2AzelUW8sqW5VGMx54dOuqO2Cs=258)). If published, this will include your full peer review and any attached files.


If you choose “no”, your identity will remain anonymous but your review may still be made public.


**Do you want your identity to be public for this peer review?** For information about this choice, including consent withdrawal, please see our [Privacy Policy](https://track.editorialmanager.com/CL0/https:%2F%2Fwww.plos.org%2Fprivacy-policy/1/010f019eca4de7f5-b991c2de-3b57-4734-bca3-9ff79ba9dd7f-000000/XE37JVcaNWyHupxFuKGD1yo0PoQCXmrGuNhSjQe26qI=258).

Reviewer #1: No

Reviewer #2: No
