# Supplementary material for: A target product profile and roadmap for Japanese encephalitis diagnostics in endemic areas in the context of other flaviviruses
Source: PLoS Negl Trop Dis. 2026 Sep 8;20(9):e0014683. doi: 10.1371/journal.pntd.0014683 (PMC13581209; doi:10.1371/journal.pntd.0014683)
Supplement: Peer review history

# Peer review: Decision Letter 0

**DOI:** 10.1371/journal.pntd.0014683.r001

**Authors:** Richard Bowen, Elvina Viennet

15 Jun 2026

PNTD-D-26-00564

Full title A target product profile and roadmap for Japanese encephalitis diagnostics in endemic areas, in the context of other flaviviruses

PLOS Neglected Tropical Diseases

Dear Dr. Bharucha,

Thank you for submitting your manuscript to PLOS Neglected Tropical Diseases. After careful consideration, we feel that it has merit but does not fully meet PLOS Neglected Tropical Diseases's publication criteria as it currently stands. Therefore, we invite you to submit a revised version of the manuscript that addresses the points raised during the review process.

Please submit your revised manuscript within by Aug 14 2026 11:59PM. If you will need more time than this to complete your revisions, please reply to this message or contact the journal office at plosntds@plos.org. When you're ready to submit your revision, log on to https://www.editorialmanager.com/pntd/ and select the 'Submissions Needing Revision' folder to locate your manuscript file.

Please include the following items when submitting your revised manuscript:

* A letter that responds to each point raised by the editor and reviewer(s). You should upload this letter as a separate file labeled 'Response to Reviewers'. This file does not need to include responses to any formatting updates and technical items listed in the 'Journal Requirements' section below.

* A marked-up copy of your manuscript that highlights changes made to the original version. You should upload this as a separate file labeled 'Revised Manuscript with Track Changes'.

* An unmarked version of your revised paper without tracked changes. You should upload this as a separate file labeled 'Manuscript'.

If you would like to make changes to your financial disclosure, competing interests statement, or data availability statement, please make these updates within the submission form at the time of resubmission. Guidelines for resubmitting your figure files are available below the reviewer comments at the end of this letter.

As the corresponding author, your ORCID iD is verified in the submission system and will appear in the published article. PLOS supports the use of ORCID, and we encourage all coauthors to register for an ORCID iD and use it as well. Please encourage your coauthors to verify their ORCID iD within the submission system before final acceptance, as unverified ORCID iDs will not appear in the published article. Only the individual author can complete the verification step; PLOS staff cannot verify ORCID iDs on behalf of authors.

We look forward to receiving your revised manuscript.

Kind regards,

Richard A. Bowen, DVM PhD

Academic Editor

PLOS Neglected Tropical Diseases

Elvina Viennet

Section Editor

PLOS Neglected Tropical Diseases

Shaden Kamhawi

co-Editor-in-Chief

PLOS Neglected Tropical Diseases

orcid.org/0000-0003-4304-636XX

Paul Brindley

co-Editor-in-Chief

PLOS Neglected Tropical Diseases

orcid.org/0000-0003-1765-0002

**Additional Editor Comments (if provided):**

Your manuscript has been reviewed by 2 experts who have offered a number of suggestioins for improvement. Please evaluate the comments from reviewers, edit your manuscript appropriately and resubmit along with a detailed list of responses to all reviewer comments. Thank you.

**Journal Requirements:**

If the reviewer comments include a recommendation to cite specific previously published works, please review and evaluate these publications to determine whether they are relevant and should be cited. There is no requirement to cite these works unless the editor has indicated otherwise.

1) Please ensure that the CRediT author contributions listed for every co-author are completed accurately and in full.

At this stage, the following Authors/Authors require contributions: Tehmina Bharucha. Please ensure that the full contributions of each author are acknowledged in the "Add/Edit/Remove Authors" section of our submission form.

The list of CRediT author contributions may be found here: https://journals.plos.org/plosntds/s/authorship#loc-author-contributions

2) Please provide an Author Summary. This should appear in your manuscript between the Abstract (if applicable) and the Introduction, and should be 150-200 words long. The aim should be to make your findings accessible to a wide audience that includes both scientists and non-scientists. Sample summaries can be found on our website under Submission Guidelines:

https://journals.plos.org/plosntds/s/submission-guidelines#loc-parts-of-a-submission

3) Your manuscript is missing the following sections: Results.  Please ensure all required sections are present and in the correct order. Make sure section heading levels are clearly indicated in the manuscript text, and limit sub-sections to 3 heading levels. An outline of the required sections can be consulted in our submission guidelines here:

https://journals.plos.org/plosntds/s/submission-guidelines#loc-parts-of-a-submission

4) Thank you for including an Ethics Statement for your study. Please include:

i) A statement that formal consent was obtained (must state whether verbal/written) OR the reason consent was not obtained (e.g. anonymity). NOTE: If child participants, the statement must declare that formal consent was obtained from the parent/guardian.].

5) Please upload all main figures as separate Figure files in .tif or .eps format. For more information about how to convert and format your figure files please see our guidelines:

https://journals.plos.org/plosntds/s/figures

6) We have noticed that you have uploaded Supporting Information files, but you have not included a list of legends. Please add a full list of legends for your Supporting Information files after the references list.

7) Please amend your detailed Financial Disclosure statement. This is published with the article. It must therefore be completed in full sentences and contain the exact wording you wish to be published.

i) State what role the funders took in the study. If the funders had no role in your study, please state: "The funders had no role in study design, data collection and analysis, decision to publish, or preparation of the manuscript."

ii) If any authors received a salary from any of your funders, please state which authors and which funders..

If you did not receive any funding for this study, please simply state: u201cThe authors received no specific funding for this work.u201d

**Reviewers' Comments:**

Reviewer's Responses to Questions

**Key Review Criteria Required for Acceptance?**

As you describe the new analyses required for acceptance, please consider the following:

**Methods**

-Are the objectives of the study clearly articulated with a clear testable hypothesis stated?

-Is the study design appropriate to address the stated objectives?

-Is the population clearly described and appropriate for the hypothesis being tested?

-Is the sample size sufficient to ensure adequate power to address the hypothesis being tested?

-Were correct statistical analysis used to support conclusions?

-Are there concerns about ethical or regulatory requirements being met?

Reviewer #1: Delphi methodology and consensus analyses were appropriately conducted and presented. Study participants were representative of JEV endemic areas and all attempts made to be inclusive of all areas impacted by JEV infection. Limitations to participant engagement was described appropriately.

Reviewer #2: The study objectives are articulated with a feasible hypothesis.

The study design looks appropriate to prepare a regional target product profile for Japanese Encephalitis. However, a clear organization is needed in the Methods Section as the Methods are also described in the Results section. A flowchart of the TPP process should be included in the Methods section.

The first version of the JE TPP was prepared based on the interviews of 5 experts. A clear justification for the limited number of experts should be included in the manuscript. Further clarification and justification for the number of participants in the 1st survey and 2nd survey is also needed.

The study lacks of statistical analysis that can support observations.

No ethical concerns.

**********

**Results**

-Does the analysis presented match the analysis plan?

-Are the results clearly and completely presented?

-Are the figures (Tables, Images) of sufficient quality for clarity?

Reviewer #1: Some consideration on rank order of test component criteria should be made (i.e. which of the testing criteria would be critical and which would be beneficial but would not preclude further development and/or evaluation if the minimal standards highlighted in the TPP were not achieved.

The role of immunomodulatory therapies on diagnostic testing performance and applicability of testing modality should be incorporated in the TPP. The use of B cell depletion therapies for a litany of clinical ailments including multiple sclerosis, rheumatoid arthritis, lymphoma etc. can negate the utility of serological-based diagnostic testing, requiring the use of antigen or nucleic acid-based testing. Although more commonly used in areas of the world not typically impacted by JEV, use of these therapies does occur in JEV endemic areas and likely will be increasing over time. A caveat to this could be included in the tiered testing discussion that was described from addressing heterologous flavivirus circulation but could also incorporate the use of different targets based on immunomodulatory treatment, immunosuppressed patients and could be a strong consideration based on the timing post-symptom onset (i.e. highly acute samples presented with acute febrile symptomology prior to inset of neurological symptoms. Furthermore, the use of nucleic acid based testing should be considered in the context of whole blood as a sample testing matric for which a series of flaviviruses have demonstrated efficient detection of viral RNA over extended time periods.

The concept of improved serological specificity through IgG depletion is not addressed in the future considerations section. Given its potential importance as part of the two-tiered testing description for addressing specific flavivirus diagnoses, I would suggest the be highlighted in the future assay considerations section.

Reviewer #2: The results presented require a clear description. For example, the criteria for concordance, feedback ratios, the number of participants (“some felt”), and criteria for too low sensitivity/specificity should be included in the text.

The Consensus building on TPP characteristics’ lack ratios or % in the text. Percentage of comments out of 44 experts that refer to the use of a test of patient management and POC is missing.

The results are not clearly presented, as certain methods (overview of the process) and redundant information are included in the Results sections.

Figures have enough quality for clarity. Flowcharts for the JE diagnostic should be included in the text. Citation of the figures in the text (E.g. Fig 1), and supplementary figures should follow PLOS guidelines.

**********

**Conclusions**

-Are the conclusions supported by the data presented?

-Are the limitations of analysis clearly described?

-Do the authors discuss how these data can be helpful to advance our understanding of the topic under study?

-Is public health relevance addressed?

Reviewer #1: Conclusions are supported by the data presented. The authors interpret findings with caution and indicate complexity appropriately. Some clarification on how clinical presentation of neurological syndromes from heterologous flaviviruses might be impacted by lack of a specific diagnosis given the largely supportive care and lack of treatment options. Heterologous flaviviruses that could also elicit neurological symptoms could included West Nile/Kunjin , Kyasanur Forest, tick-borne encephalitis, Murray Valley encephalitis and Zika viruses.

The discussion points should be made in the context of what sample matrices would be optimal for disease presentation differences and time post symptom onset. There is no mention of the potential diagnostic utility of IgA testing

Reviewer #2: Conclusions are partially supported by the data presented. Data results must be clearly presented and organized in the manuscript. The study mentioned current issues with the JE diagnostic and the consideration of new diagnostic tests.

The authors discussed how the TPP for JE has highlighted several topics for JEV that require further research and attention.

Public health relevance of the Target product profile for Japanese encephalitis is addressed.

**********

**Editorial and Data Presentation Modifications?**

Use this section for editorial suggestions as well as relatively minor modifications of existing data that would enhance clarity. If the only modifications needed are minor and/or editorial, you may wish to recommend “Minor Revision” or “Accept”.

Reviewer #1: The authors use the term “point-of-care test”; however, a descriptor of “rapid diagnostic test” would be a more appropriate term that would encapsulate the different attributes highlighted in the target product profile given that some of the benefits would be surveillance-oriented as well as direct care might not be available and assessment of alternative etiologies would be sought or patients would be either enrolled in JEV clinical treatment trials or admission to neuro-intensive care unit more efficiently.

Introduction line 135: some mention of whether Culex control is utilized as a public health measure in response to documented human clinical disease cases.

Line 230-Overiew of the Process: should this description be included in the methods section as opposed top the description of the findings?

Table 1: Test procedures- the topic of multiplexing for the assessment (i.e. rule-out) of heterologous circulating flaviviruses is described in the context of two-tiered testing. Some description of the relative impact that multiplex testing can have on assay performance standards should be included (i.e. principally, the negative impact on test sensitivity).

Table 1: Ease of Use- a determination of a minimal standard for four steps should be presented in the context of non-inferiority of current diagnostics assays in use.

Table 1: Test kit stability/operational conditions- there is no consideration of how the shipment and storage of testing components could be augmented by the use of desiccants. The impact on nucleic acid testing has been demonstrated to be extended on FTA cards in which desiccants have been utilized.

Table 1: kit controls- some discussion of sample viability determination should be included (determination of the level of hemolysis, presence of inhibitors etc.) should be considered.

Line 320: a reference for the 10% diagnostic testing rate for NS1/RNA. As described above this would be mandated for immunosuppressed patients.

Line 331: “RT-PCR”

Table 2: given the presentation of product development consideration and test implementation in the table (in that order), the title should mention in the same order.

Reviewer #2: (No Response)

**********

**Summary and General Comments**

Use this section to provide overall comments, discuss strengths/weaknesses of the study, novelty, significance, general execution and scholarship. You may also include additional comments for the author, including concerns about dual publication, research ethics, or publication ethics. If requesting major revision, please articulate the new experiments that are needed.

Reviewer #1: The PLoS Neglected Tropical Diseases Manuscript PNTD-D-26-00564 entitled “A target product profile and roadmap for Japanese encephalitis diagnostics in endemic areas, in the context of other flaviviruses” by Bharucha et al. describes the results of a Delphi process that was utilized to refine the content of target product profile for Japanese encephalitis virus. The investigators identified a consensus on the majority of the components for minimal and ideal performance standards for attributes of the JEV diagnostic test. Emphasis of performance characteristics for which diagnostic testing would be performed as a priority for patient management was relayed by on the overwhelming consensus of participants that this should be the testing priority. The authors should be commended on the thorough addressing a very complicated topic that addresses diagnostic challenges in the context of circulating heterologous flaviviruses that complicates serodiagnosis as well as the historical emphasis on vaccine distribution at the expense of improved JEV diagnostics. General and specific comments for consideration by the authors have been provided.

Reviewer #2: Japanese encephalitis (JE) virus has been one of the most important aetiologic agents of severe neurological infections in Asia and the Pacific regions. The study aimed to develop, for the first time, a regional target product profile (TPP) for JE diagnostics in endemic areas to improve patient management and public health. Initial interviews were performed with five selected experts to prepare a first version of a TPP document. Then, a Delphi methodology was used to obtain consensus from a subgroup of 9.56% (44/460) experts, aiming for≥75% consensus on each JE test characteristic described in the TPP, through two online surveys. Subsequently, a one-day meeting facilitated in-depth discussion with 42 out of 44 participant experts. Overall, seven priority JE characteristics were discussed, and consensus was achieved for all characteristics, with the exception of the preferred JE test sensitivity and specificity (73% agreement). The TPP for the JE test outlines a critical characteristic for future development and implementation in the region. However, the manuscript requires a major revision for further publication consideration.

The abstract requires to integrate and summarize the methods and results of the study. A broad impact of the TPP study should be mentioned as the final sentence, Furthermore, the manuscript requires more attention in the methods and results section, including figures and supplementary data. Flowcharts for the TPP process and algorithm should be included in the manuscript. Grammar throughout the text needs to be revised.

Specific comments:

Line 79-99, Revise and integrate the abstract

Line 111-112, Revise sentence “approximately 15-30% die”

Line 122, Spell out 16/25

Line 143, Add the words “serotypes 1 to 4” in parenthesis

Line 145-145, Revise sentence “is even lower “

Line 148, Replace “titer” with “test”

Line 167, Is “AND diagnos” correct?

Line 186, Suggest including the word” preliminary draft”

Line 190, Describe JE characteristics

Line 217, Provide the name, degree, and affiliation of Dr. Ava Easton

Line 271-272, justify the use of the word “too low” in the text

Line 274, Suggest including the word “revised TPP”

Line 283-5, Cite Figures in the text by using the word “Fig.”

Line 305, Spell out “RDT”

Line 335-338, revise the sentence

Line 347, revise sentence “we see expansion of JEV into new areas, and ongoing cases in areas that are vaccinating”

Line 350-351, revise sentence “The scoping interviews reiterated the appetite for this work”

Line 373, Spell out “9/25”

Table 1. Revise grammar

**********

PLOS authors have the option to publish the peer review history of their article (what does this mean?). If published, this will include your full peer review and any attached files.

If you choose “no”, your identity will remain anonymous but your review may still be made public.

**Do you want your identity to be public for this peer review?**  For information about this choice, including consent withdrawal, please see our Privacy Policy.

Reviewer #1: No

Reviewer #2: No

[NOTE: If reviewer comments were submitted as an attachment file, they will be attached to this email and accessible via the submission site. Please log into your account, locate the manuscript record, and check for the action link "View Attachments". If this link does not appear, there are no attachment files.]

**Figure resubmission:**

**Reproducibility:**

To enhance the reproducibility of your results, we recommend that authors of applicable studies deposit laboratory protocols in protocols.io, where a protocol can be assigned its own identifier (DOI) such that it can be cited independently in the future. Additionally, PLOS ONE offers an option to publish peer-reviewed clinical study protocols. Read more information on sharing protocols at https://plos.org/protocols?utm_medium=editorial-email&utm_source=authorletters&utm_campaign=protocols

# Author comment: Author response to Decision Letter 1

**DOI:** 10.1371/journal.pntd.0014683.r002

29 Jul 2026

Attachment Submitted filename: Response to Reviewers.docx

# Peer review: Decision Letter 1

**DOI:** 10.1371/journal.pntd.0014683.r003

**Authors:** Richard Bowen, Elvina Viennet

17 Aug 2026

Dear Dr Bharucha,

We are pleased to inform you that your manuscript 'Full title A target product profile and roadmap for Japanese encephalitis diagnostics in endemic areas, in the context of other flaviviruses' has been provisionally accepted for publication in PLOS Neglected Tropical Diseases.

Before your manuscript can be formally accepted you will need to complete some formatting changes, which you will receive in a follow up email. A member of our team will be in touch with a set of requests.

Please note that your manuscript will not be scheduled for publication until you have made the required changes, so a swift response is appreciated.

IMPORTANT: The editorial review process is now complete. PLOS will only permit corrections to spelling, formatting or significant scientific errors from this point onwards. Requests for major changes, or any which affect the scientific understanding of your work, will cause delays to the publication date of your manuscript.

Should you, your institution's press office or the journal office choose to press release your paper, you will automatically be opted out of early publication. We ask that you notify us now if you or your institution is planning to press release the article. All press must be co-ordinated with PLOS.

Thank you again for supporting Open Access publishing; we are looking forward to publishing your work in PLOS Neglected Tropical Diseases.

Best regards,

Richard A. Bowen, DVM PhD

Academic Editor

PLOS Neglected Tropical Diseases

Elvina Viennet

Section Editor

PLOS Neglected Tropical Diseases

Shaden Kamhawi

co-Editor-in-Chief

PLOS Neglected Tropical Diseases

orcid.org/0000-0003-4304-636XX

Paul Brindley

co-Editor-in-Chief

PLOS Neglected Tropical Diseases

orcid.org/0000-0003-1765-0002

***********************************************************

Thank you for the careful revision to your manuscript. This is a valuable contribution and now acceptable for publication in PLoS NTD.

p.p1 {margin: 0.0px 0.0px 0.0px 0.0px; line-height: 16.0px; font: 14.0px Arial; color: #323333; -webkit-text-stroke: #323333}span.s1 {font-kerning: none

Reviewer's Responses to Questions

**Key Review Criteria Required for Acceptance?**

As you describe the new analyses required for acceptance, please consider the following:

**Methods**

-Are the objectives of the study clearly articulated with a clear testable hypothesis stated?

-Is the study design appropriate to address the stated objectives?

-Is the population clearly described and appropriate for the hypothesis being tested?

-Is the sample size sufficient to ensure adequate power to address the hypothesis being tested?

-Were correct statistical analysis used to support conclusions?

-Are there concerns about ethical or regulatory requirements being met?

Reviewer #1: authors have addressed critique sufficiently.

Reviewer #2: The questions regarding the methods were addressed by the authors.

**********

**Results**

-Does the analysis presented match the analysis plan?

-Are the results clearly and completely presented?

-Are the figures (Tables, Images) of sufficient quality for clarity?

Reviewer #1: authors have addressed critique sufficiently.

Reviewer #2: The results are now clearly presented.

**********

**Conclusions**

-Are the conclusions supported by the data presented?

-Are the limitations of analysis clearly described?

-Do the authors discuss how these data can be helpful to advance our understanding of the topic under study?

-Is public health relevance addressed?

Reviewer #1: authors have addressed critique sufficiently.

Reviewer #2: The conclusions are supported by the data presented, clearly describing their limitation and future directions.

**********

**Editorial and Data Presentation Modifications?**

Use this section for editorial suggestions as well as relatively minor modifications of existing data that would enhance clarity. If the only modifications needed are minor and/or editorial, you may wish to recommend “Minor Revision” or “Accept”.

Reviewer #1: authors have addressed critique sufficiently.

Reviewer #2: (No Response)

**********

**Summary and General Comments**

Use this section to provide overall comments, discuss strengths/weaknesses of the study, novelty, significance, general execution and scholarship. You may also include additional comments for the author, including concerns about dual publication, research ethics, or publication ethics. If requesting major revision, please articulate the new experiments that are needed.

Reviewer #1: authors have addressed critique sufficiently.

Reviewer #2: The manuscript developed a target product profile for the diagnosis of Japanese encephalities, which is critical for future development and implementation in endemic regions.

**********

PLOS authors have the option to publish the peer review history of their article (what does this mean?). If published, this will include your full peer review and any attached files.

If you choose “no”, your identity will remain anonymous but your review may still be made public.

**Do you want your identity to be public for this peer review?**  For information about this choice, including consent withdrawal, please see our Privacy Policy.

Reviewer #1: No

Reviewer #2: No

# Editor report: Acceptance letter

**DOI:** 10.1371/journal.pntd.0014683.r004

**Authors:** Richard Bowen, Elvina Viennet

Dear Dr Bharucha,

We are delighted to inform you that your manuscript, "Full title A target product profile and roadmap for Japanese encephalitis diagnostics in endemic areas, in the context of other flaviviruses," has been formally accepted for publication in PLOS Neglected Tropical Diseases.

We have now passed your article onto the PLOS Production Department who will complete the rest of the publication process. All authors will receive a confirmation email upon publication.

The corresponding author will soon be receiving a typeset proof for review, to ensure errors have not been introduced during production. Please review the PDF proof of your manuscript carefully, as this is the last chance to correct any scientific or type-setting errors. Please note that major changes, or those which affect the scientific understanding of the work, will likely cause delays to the publication date of your manuscript. Note: Proofs for Front Matter articles (Editorial, Viewpoint, Symposium, Review, etc...) are generated on a different schedule and may not be made available as quickly.

Soon after your final files are uploaded, the early version of your manuscript will be published online unless you opted out of this process. The date of the early version will be your article's publication date. The final article will be published to the same URL, and all versions of the paper will be accessible to readers.

For Research Articles, you will receive an invoice from PLOS for your publication fee after your manuscript has reached the completed accept phase. If you receive an email requesting payment before acceptance or for any other service, this may be a phishing scheme. Learn how to identify phishing emails and protect your accounts at https://explore.plos.org/phishing.

Thank you again for supporting open-access publishing; we are looking forward to publishing your work in PLOS Neglected Tropical Diseases.

Best regards,

Shaden Kamhawi

co-Editor-in-Chief

PLOS Neglected Tropical Diseases

Paul Brindley

co-Editor-in-Chief

PLOS Neglected Tropical Diseases
